# How Geometric Constraints Control the Hydride Position and Activity in [NiFe]-Hydrogenases and Their Biomimetic Complexes

**DOI:** 10.1021/acs.inorgchem.5c00670

**Published:** 2025-05-09

**Authors:** Shuqiang Niu, Michael B. Hall

**Affiliations:** Department of Chemistry, Texas A&M University, College Station, Texas 77843-3257, United States

## Abstract

The **Ni-R** active site in [NiFe]-hydrogenase features a bridging hydride between the Ni and Fe, displaced toward the Ni. However, all synthetic **Ni-R** models reported to date exhibit a hydride displaced toward Fe and display low turnover frequencies for H_2_ evolution. Understanding the factors governing the hydride position and activity of **Ni-R** and biomimetic complexes is crucial for developing efficient hydrogen-evolving catalysts. By utilizing the CCSD theory, DFT, NBO, and QTAIM analysis, we investigated these factors in a **Ni-R** active-site model (**1**), and two representative biomimetic complexes, 2* and **3**. Our results reveal that the Ni site of **1** inherently prefers a square-planar [S_2_NiSH] configuration with an apically positioned thiolate and that hydride positioning is governed by the strength of [Ni–H–Fe] three-center two-electron bonding, which is modulated by the geometric torsion between the Ni terminal ligands and the bridging thiolates. By modifying the linkers between the Ni terminal ligands and bridging thiolate ligands of 2* and **3**, we designed virtual biomimetic complexes (**4–10**). These complexes exhibit improved hydride nucleophilicity and increased potential for H_2_ formation, providing valuable insights into how geometric and electronic factors influence hydride activity and informing the design of more effective biomimetic hydrogenase models.

## Graphical abstract

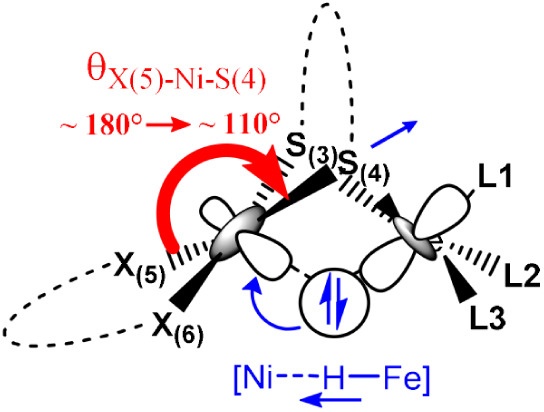

## Introduction

[NiFe]-hydrogenases ([NiFe]-H_2_ase) are enzymes that facilitate the reversible conversion between H_2_ and protons/electrons under mild conditions,^1,2^ making them ideal models for studying how nature developed hydrogen consuming and evolving catalysts (HECs) and for bioengineering and biomimetic approaches to hydrogen production, storage, and utilization.^3,4^ Extensive theoretical^5–20^ and experimental^2,21–30^ investigations of [NiFe]-H_2_ase have led to the general acceptance of **Ni-SI**_**a**_, **Ni-C**, and **Ni-R** species as intermediates in the catalytic cycle of hydrogen evolution (the top circle of Scheme 1). The **Ni-R** active site, [(CO)­(CN)_2_Fe­(μ-S_cys_)_2_(μ-H)­Ni­(HS_cys_)­(S_cys_)]^2–^ (S_cys_ = cysteine), which is characterized by a bridging hydride between the Ni and Fe centers but displaced toward the Ni site,^21,31^ plays a critical role from hydrogen evolution to utilization.^11^ However, synthetic biomimetic complexes of the **Ni-R** model (**1**) reported thus far, such as [(dppe)­Ni­(μ-pdt)­(μ-H)­Fe­(CO)_3_]^+^ (2*, pdt = 1,3-propanedithiolate; dppe = 1,2-C_2_H_4_(PPh_2_)_2_) and [Ni­(N2S2)**·**FeCp]^+^ (**3**, N2S2 = bismercaptoethane diazacycloheptane, Cp = η^5^-C_5_H_5_^–^) (Scheme 1, diamond at the bottom), exhibit the hydride shifted toward or bound mainly to Fe (Table S1),^4,13,32–47^ deviating from the **Ni-R** active site, and lower turnover frequencies (TOF) for H_2_ evolution.^13,32,38–40^ Unraveling how variations in the geometric and electronic properties of the Ni and Fe ligands in the **Ni-R** active site and the biomimetic complexes influence hydride positioning and activity may aid synthetic efforts in accelerating the development of biomimetic catalysts for hydrogen evolution.

**Scheme 1:**
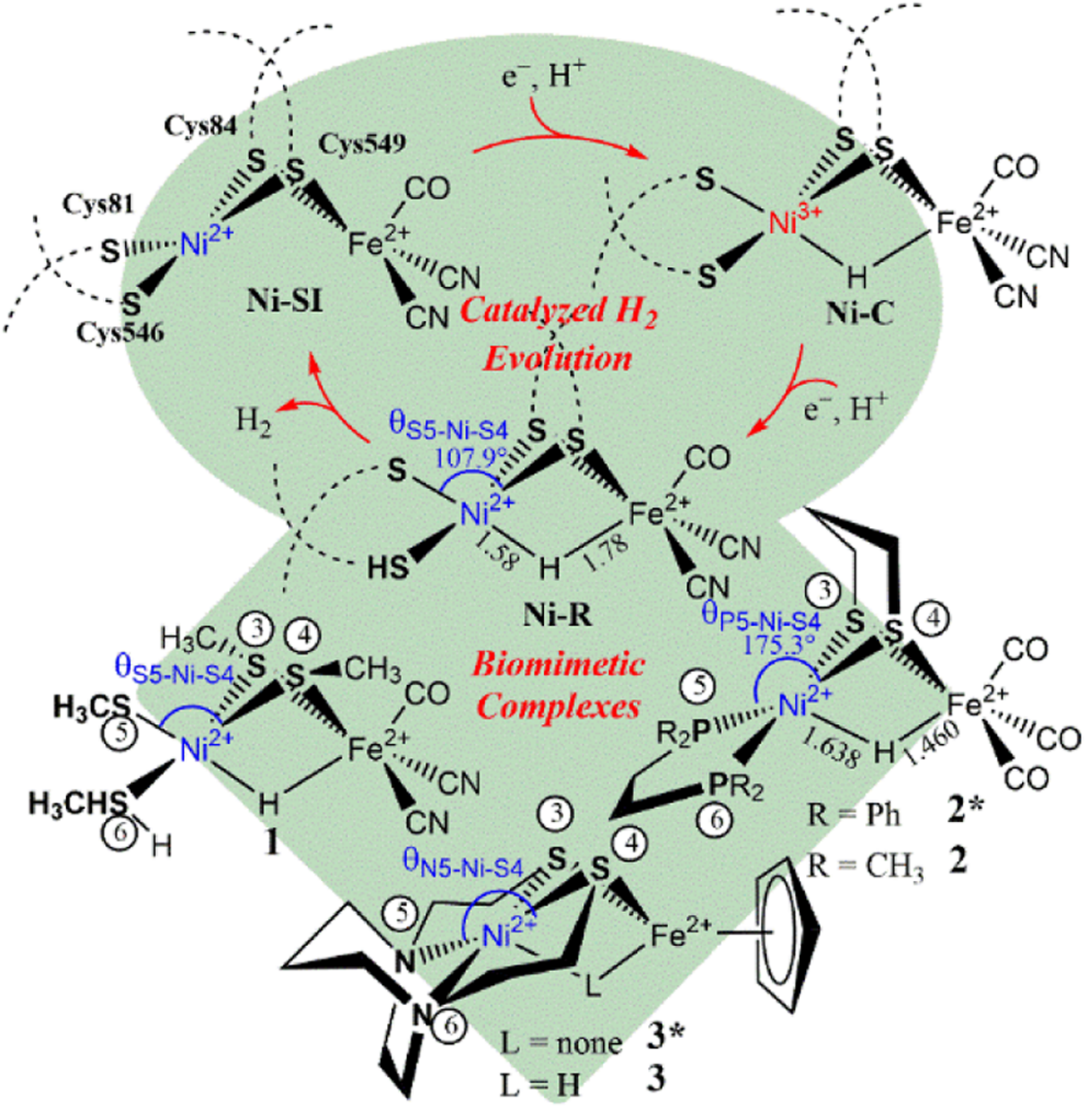


The high-resolution X-ray crystal structure of [NiFe]-H_2_ase as **Ni-R** from D. vulgaris *Miyazaki F* protein revealed that the [S_2_NiS_2_] (S = sulfur of cysteines) framework and the bridging hydride form a nearly square pyramidal structure about the Ni with the hydride, Cys81, Cys84, and Cys546 in basal positions, and Cys549 in an apical position.^21^ The Fe site coordinates with one CO and two CN ligands and bridges to the hydride and the two cysteines (Cys84 and Cys549 bridging to Ni), which results in a nearly octahedral Fe geometry (Scheme 1). Overall, this arrangement leads to a longer Ni–Cys549 distance of 2.54 Å compared to the average of 2.21 Å for the other Ni–Cys bond lengths. Notably, the Ni–H bond length in **Ni-R** measures 1.58 Å, which is 0.20 Å shorter than the Fe–H bond length of 1.78 Å.

Theoretical studies have suggested both heterolytic and homolytic mechanisms for the reversible conversion of molecular hydrogen in [NiFe]-H_2_ase.^6,11,48^ In the heterolytic mechanism, the H–H bond is formed via a nucleophilic addition pathway, where the bridging nucleophilic hydride (H^–^) and the electrophilic proton (H^+^) on Cys546 approach each other to form H_2_, and the H–H bond is cleaved by the reverse reaction. In the homolytic mechanism, the H–H bond is formed/cleaved via a reductive-elimination/oxidation-addition pathway. Here, the proton on Cys546 is transferred to the Ni­(II) site, forming a dihydride Ni­(IV) intermediate, which then releases H_2_ by coupling the two H^•^ radicals. Therefore, the displacement of the bridging hydride toward the Ni in **Ni-R** is likely conducive to the H_2_ formation by either reaction mechanism.

Recent advancements have been made in the development of synthetic models of the [NiFe]-H_2_ase active site, specifically mimicking the [Ni­(μ-S)_2_(μ-H)­Fe] core found in the enzymes.^4,13,29,32–47,49–51^ These biomimetic complexes reported in the literature have shown similar structural characteristics, with the hydride positioned apically to the square-planar [X_2_NiS_2_] (X = N, P) moiety and in closer proximity to the Fe atom. In summary, the Ni–H and Fe–H bond lengths in these complexes range from 1.64 to 2.16 Å and 1.46 to 1.65 Å, respectively (see Table S1),^32–37^ and their TOF for H_2_ evolution are low.^32,40^ In particular, the biomimetic complex, 2* (Scheme 1),^32^ exhibits a square pyramidal structure on the Ni site with hydride in the apical site with a Ni–H bond length of 1.64 Å and a short Fe–H bond of 1.46 Å. Additionally, several biomimetic complexes containing metallodithiolate [N_2_NiS_2_] moiety bound to CpFe or Fe­(NO)_2_, including 3* (Scheme 1), have been recently reported and have demonstrated modest electrocatalysis and H_2_ production capabilities.^43,44,46,51^ Experimental and theoretical studies supported the existence of a hydride intermediate like **3** (Scheme 1), again with a long Ni–H distance, in their hydrogen evolution reaction.^36–38,45,46^

To investigate how variations in the geometric and electronic properties of the Ni terminal ligands influence the hydride position and activity of the **Ni-R** and the biomimetic complexes 2* and 3*, we performed comprehensive density functional theory (DFT)^52^ and coupled-cluster singles-and-doubles (CCSD)^53^ calculations, along with natural bond orbital (NBO)^54^ and quantum theory of atoms in molecules (QTAIM)^55^ analyses. We begin by examining the factors that contribute to stability of the [S_2_Ni­(μ-S)_2_(μ-H)­Fe] core in **Ni-R**. Next, we identify the factors controlling the hydride position and activity relevant to H_2_ evolution. Finally, based on these key factors (descriptors), we computationally generate a series of analog complexes of the biomimetic complexes 2* and 3* with the desired structural and electronic properties optimized to resemble those of **Ni-R**.

## Computational Methods

The major model complexes studied are [(HSMe)­(SMe)­Ni­(μ-SMe)_2_(μ-H)­Fe­(CO)­(CN)_2_]^2–^ (**1**, where Me = CH_3_), (dmpe)­Ni­(μ-pdt)­(μ-H)­Fe­(CO)_3_]^+^ [**2**, where dmpe= 1,2-bis­(dimethylphosphino)­ethane], and [Ni­(N2S2)­(μ-H)**·**FeCp]^+^ (**3**) (Scheme 1). Full and constraint geometry optimizations of complex **1**, a model of the **Ni-R** active site, were performed using CCSD and DFT methods. In the CCSD calculations, the 6-31G** basis sets were employed for the Ni, Fe, S, and hydrogens involved in the H_2_ formation/cleavage, while the STO-3G basis sets were utilized for methyl, CO, and CN groups. The frozen-core approximation was applied, in which all valence electrons were correlated, including the Ni and Fe 3s and 3p electrons. In the DFT calculations, spin-unrestricted DFT approach was performed with extra fine grids for numerical integration (target accuracy of 10^–8^ au in energy calculations). Five functionals,^52,56^ BP86, TPSS, B3LYP, M06, and LC-ωPBEh, with two basis sets, DZVP2 and Def2-TZVP, were assessed for their accuracy in determining geometric and energetic properties of **Ni-R** active site model complex **1** (Tables S2 and S3).

Previous studies have demonstrated the effectiveness of the M06/DZVP2 approach in accurately describing geometries, inner-sphere reduction energies, thermodynamic and kinetic properties of redox and catalytic sites in various first-row transition-metal systems like bimetallic hydrogenases,^57^ iron–sulfur electron transfer proteins,^58^ blue-copper proteins,^59^ and [M­(mnt)_2_]^*n*−^ (M = Ni, Fe; mnt = 1,2-S_2_C_2_(CN)^2–^; *n* = 2, 1) (see Figure S1). Overall, the M06/DZVP2 (or M06/DGDZVP2) offered a reliable balance of computational cost and accuracy for modeling geometries and energies of first-row transition-metal systems complexes. Therefore, M06/DZVP2 was primarily utilized for geometry optimizations and energy calculations in this study.

To account for the solvation free energy of the protein environments (ε ≈ 4.0) on the structure of the **Ni-R** active site, the full geometry optimizations were also carried out on **1** in the diethylamine solution phase (ε = 3.6) utilizing the SMD continuum solvation model.^60^ The relative energies (Δ*E*_e_) and the relative free energy (Δ*G*) were determined using the total electronic energy (*E*_e_) and Gibbs free energy (*G*) of molecules, calculated at either fully optimized or constrained optimized geometries. Gibbs free energies (*G* = *H* – *TS*) at temperature of 298.15 K were obtained by summing the calculated *E*_e_ and thermal energy correction to G. The thermal energy correction to enthalpy (*H*_corr_ = ZPE + *E*_vib_ + *E*_rot_ + *E*_rot_+ *RT*) and the total entropy (*S*) were derived from a frequency calculation. Additionally, the hydride bonding energies (Δ*G*_H^–^_) of the complexes (MH^–^) in the MeCN solution were calculated at the M06/DZVP2 level and are defined as

$\Delta G_{H^{-}}=G({\mathrm{MH}}^{-})-[G(M)+G(H^{-})]$

The QTAIM^55^ and NBO^54^ analyses were employed to evaluate the atomic charges on the atom A (*Q*_A_), the electron delocalization indexes of an A–B bond (δ_A–B_), the orbital occupancy *Q*_Ni–H–Fe_, orbital energy *E*ψ_Ni–H–Fe_, and NBO stabilization energies *E* ^2^ of donor–acceptor interactions (using second-order perturbation theory).

All DFT calculations were conducted using the NWChem software packages.^61^ The G09 computational chemistry software package^62^ was utilized for CCSD and NBO calculations while the QTAIM calculations were performed with the AIMALL software package.^63^ Structural and electronic structural visualizations and analysis were carried out using the GaussView,^62^ ChemOffice,^64^ Molden,^65^ and Chimrea.^66^ The simulated reaction pathways were visualized and converted into videos using GaussView, with the IRC (intrinsic reaction coordinate) was generated by our mLST-IRC program. The mLST-IRC program generates reaction trajectories by linking key points, such as reactants, intermediates, transition states, and products, using multilinear synchronous transit (mLST) calculations (see Supporting Information).

## Results and Discussion

To understand the unusual structural characteristics of the **Ni-R** active site, we conducted detailed calculations on the model complex **1** at various levels of theory. Initially, DFT calculations of the model complex **1** in the gas phase utilizing various functionals and basis sets, ranging from the pure-GGA through hybrid/meta GGA to long-range corrected functionals, revealed a square-planar [S_2_NiSH] site with the apical fourth thiolate with a longer Ni–S distance bridging to the Fe. Specifically, for all the methods, the fully optimized structure **1a** of the model complex **1**, exhibited similar bond lengths except that of the apical Ni–S(4), which displayed a wider range of distances (Table S2). The DFT results are also consistent with those optimized at the CCSD/6-31G** level of theory and, aside from this longer Ni–S(4) [Ni–S_(Cys549)_] distance, all the computed distances are in good agreement with the experimental values for **Ni-R** (Table S2).

Next, to assess the electrostatic effects of protein environments on the Ni–S_(Cys549)_ distance, we performed the full geometry optimization for **1a** in solution (diethylamine with ε = 3.6, like protein environments of ∼4). The slight decrease of 0.008 Å in the apical Ni–S(4) distance between the gas phase and solution phase suggests that the shorter Ni–S_(Cys549)_ bond in **Ni-R** compared to **1a** is likely due to the structural influence of protein backbone. Finally, the constraint-optimized geometric parameters of the model complex **1a1**, obtained through the DFT calculations with a fixed bridging Ni–S(4) distance of 2.54 Å, like that in **Ni-R**, show excellent agreement with the active site of **Ni-R** (Table S3). For instance, the RMSD (Root Mean Square Deviation) of the calculated geometry at the M06/DZVP2 level of theory for **1a1** compared to the active site of **Ni-R** is 0.250 Å (Figure S2a). In particular, the calculated θ_S(5)–Ni–S(4)_ angle of 114.9°, the Ni–H, Fe–H, and Ni–S(3) bond lengths of 1.59 Å, 1.67 Å, and 2.21 Å for **1a1** at the M06/DZVP2 level closely match the experimental values of 107.9°, 1.58 Å, 1.78 Å, and 2.21 Å for the active site of **Ni-R** (Table S3), respectively. Notably, the differences in energy (Δ*E*_e_) between **1a** and **1a1** at the CCSD/6-31G** and the M06/DZVP2 levels are only 0.71 and 3.87 kcal/mol, respectively (Table S3); suggesting that the structural changes arising from variation of the Ni–Cys549 are intrinsically low energy.

Furthermore, to understand the intrinsic structural properties of biomimetic complexes and their relationship the structure of **Ni-R**, we performed full geometry optimization on a conformer **1b** of the model complex **1**. The initial geometry for **1b** featured a [Ni­(μ-S)_2_(μ-H)­Fe] core with a square-planar [S_2_NiS_2_] site, similar to the [X_2_NiS_2_] site in 2* and **3** (where X = P or N), with an apically positioned hydride. While attempts to optimize **1b** in the gas phase lead back to **1a** (Video S1), the geometry optimization of **1b** in the solution phase at the M06/DZVP2 level yielded a structural profile similar to those of 2* and **3** with the [S_2_NiS_2_] nearly square planar and the H in the apical site, where the Ni–S, Ni–H, and Fe–H bond distances of **1b** are 2.20 Å, 1.960 Å and 1.662 Å, respectively. The relative energy (Δ*E*_e_) of 8.28 kcal/mol between **1a** and **1b** in the solution phase confirms that **1a** is a global minimum (Table S4).

Since **1b** has a structure like the biomimetic complexes, while **1a** (and especially **1a1** with the constrained Ni–S(4) distance) have structures that are nearly identical to that of **Ni-R**, we examined the influence of the Ni terminal ligand’s angular geometry on the hydride position and properties of the model complex **1** through a potential energy surface (PES) scan and QTAIM topological analysis. With the model **1a1**, the geometry optimized PES scan at the M06/DZVP2 level of theory involved increasing the angle θ_S(5)–Ni–S(4)_ from 115° (**1a1**) to 140° (**1a2**), 160° (**1a3**) and finally 180° (**1a4**, which is like **1b**), while maintaining the bridging Ni–S(4) and Ni–S(3) distances of **1a2** to **1a4** at 2.54 and 2.21 Å, respectively, as observed in **Ni-R** and **1a1**. As the θ_S(5)–Ni–S(4)_ angle increased from **1a1** to **1a4**, a significant displacement of the hydride away from the Ni toward Fe was observed, an increase of 0.32 Å in the Ni–H bond length accompanied by an increase of 0.17 Å in the Ni–Fe distance (Figure 1a, Tables 1, S4, and Video S2). The total electronic energy (*E*_e_) increased smoothly from **1a1** to **1a4** without encountering a barrier. QTAIM topological analysis (Tables 1 and S4) revealed a Ni–H electron delocalization index (δ_Ni–H_), which measures the degree of electron sharing or delocalization between the Ni and hydride in real space, of 0.54 au in **1a1**, larger than the δ_Fe–H_ of 0.41 au. As the θ_S(5)–Ni–S(4)_ angle increased from **1a1** to **1a4**, we observed a significant decrease in δ_Ni–H_ to 0.26 au and a corresponding increase in δ_Fe–H_ to 0.61 au. Additionally, the negative charge on the hydride (*Q*_H_) experienced a slight increase, which was unexpected (*vide infra*), as one might have expected the **Ni-R**-like structure, **1a1**, to be more hydridic compared to the biomimetic-like structure, **1a4**.

**Table 1:** M06/DZVP2 Geometric Parameters (In Deg and Å; X = S, P, N), NBO Properties (3c-2e Orbital Occupancy *Q*_Ni–H–Fe_ and Orbital Energy *E*ψ_Ni–H–Fe_ in Atomic Unit), QTAIM Topological Properties (Charge *Q* and Electron Delocalization Index δ in Atomic Unit), Relative Total Electron Energy Δ*E*_e_, Relative Free Energy Δ*G*, and Hydride Bonding Free Energy Δ*G*_H^–^_ (kcal/mol) for the **Ni-R** Active Site Model **1**, Biomimetic Models **2** and **3**

	**1a1** ^a^	**1a4** ^a^ ^b^	**2b4** ^b^	**2b**	**3a3** ^b^	**3a**
θ_X(5)–Ni–S(4)_	114.9	**180.0**	**105.3**	175.9	**120.0**	171.4
*r*_Ni–Fe_	2.557	2.724	2.421	2.665	2.366	2.590
*r*_Ni–H_	1.590	1.909	1.615	1.897	1.664	2.081
*r*_Fe–H_	1.669	1.603	1.674	1.588	1.630	1.581
*Q*_Ni–H–Fe_	1.74	1.77	1.72	1.73	1.83	1.88
*E*ψ_Ni–H–Fe_	–0.12297	–0.03438	–0.52572	–0.44423	–0.36280	–0.27307
*Q*_Ni_	0.392	0.401	0.166	0.190	0.515	0.544
*Q*_Fe_	0.834	0.821	0.799	0.795	0.733	0.680
*Q*_H_	–0.215	–0.298	–0.210	–0.219	–0.294	–0.373
δ_Ni–H_	0.544	0.261	0.480	0.251	0.510	0.182
δ_Fe–H_	0.408	0.609	0.465	0.625	0.542	0.731
Δ*E*_e_ ^c^	0.00	12.97	0.00	–23.11	0.00	–20.65
Δ*G* ^d^	0.00	9.08	0.00	–21.39	0.00	–24.81
Δ*G*_H^–^_ ^d^	–50.72	–30.66	–83.83	–73.03	–49.89	–28.47

a Constrained *r*_Ni– S(3)_ and *r*_Ni– S(4)_ at 2.21 Å and 2.54 Å, respectively.

b Constrained θ_S(5)–Ni–S(4)_ (in bold) in the geometry optimizations.

c In the gas phase.

d In the acetonitrile solution.

**Figure 1:**
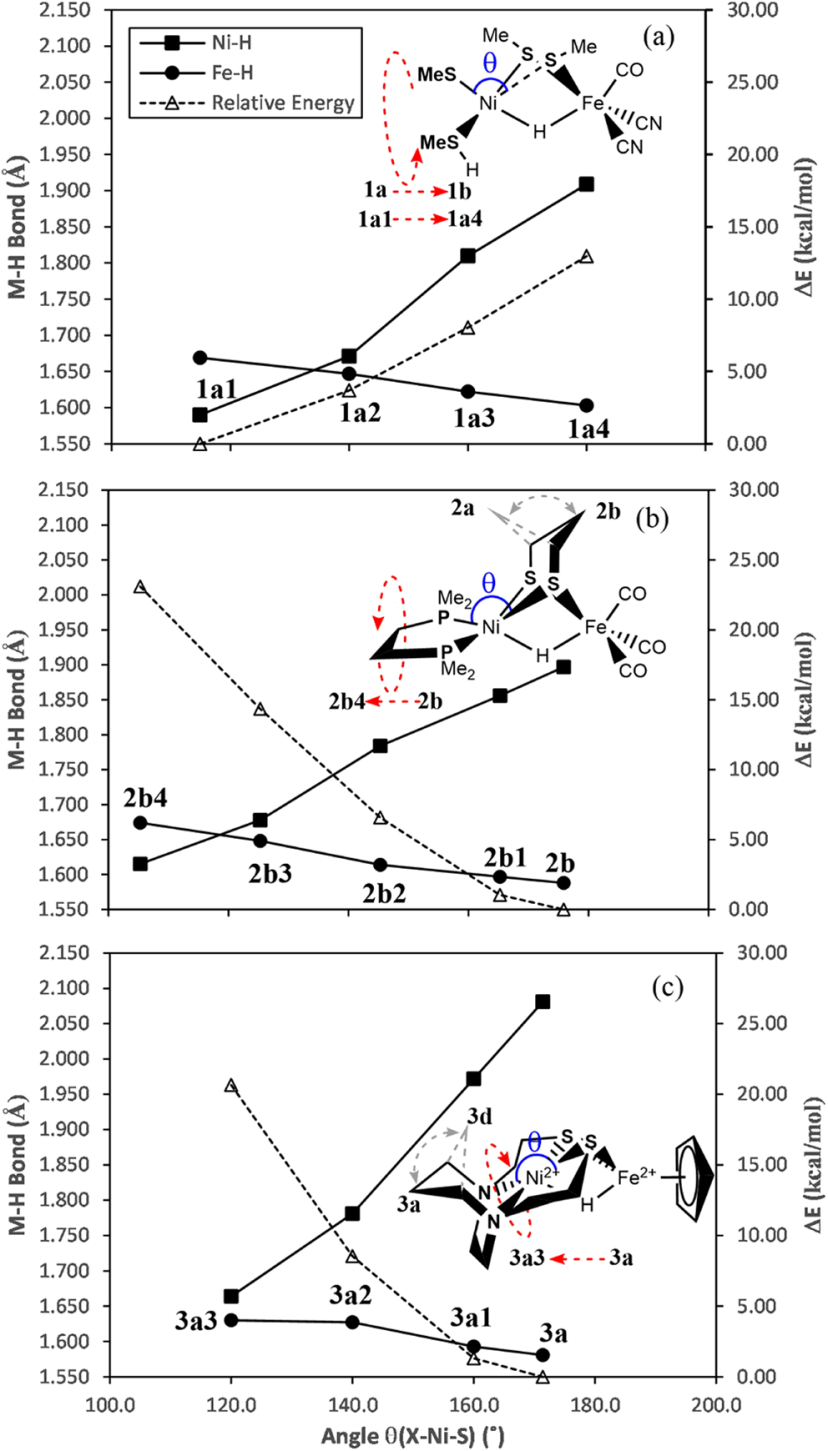
Plot of variations of the M–H bond lengths (M = Ni: solid square; M = Fe: solid circle), relative total electronic energy (Δ*E*_e_; open triangle) with the increase in the θ_X(5)–Ni–S(4)_ angle (X = S for **1**, P for **2**, N for **3**): (a) from **1a1** to **1a4**, (b) from **2b** to **2b4**, and (c) from **3a** to **3a3**, respectively. The changes in the conformational structure between **2a** and **2b** and between **3a** and **3d** are depicted by gray dashed lines.

Further molecular orbital (MO) and NBO analyses provide deeper insight into the exothermic nature and the decrease in hydridic character from **1a4** to **1a1**. The MO interaction diagram between an octahedral [FeH­(SMe)_2_(CN)_2_(CO)]^3–^ and a right triangle [(SMe)­(SMeH)­Ni]^+^ fragments for **1a4** reveals that a [Ni–H–Fe] three-center-two-electron (3c-2e) bond (ψ_Ni–H–Fe_) can be formed via interactions between the hydride orbital, which is the σ_Fe–H_ bond (H_1s_ + Fe_4pz_), and the unoccupied 4p_*z*_ and 4s orbitals of the Ni site (Figure S3). This 3c-2e bond has a longer Ni–H distance as it is destructively interacting with the doubly occupied Ni $d_{z^{2}}$ . During the transition from **1a4** to **1a1**, the overlap of the bonding orbitals between the hydride and the Ni site in this 3c-2e bond increases as the occupied Ni d_z2_ moves away. This enhanced bonding interaction leads to greater stabilization energy for the ψ_Ni–H–Fe_ orbital in **1a1** compared to **1a4** (Figure S4). As a result of the hydride shifting toward Ni, the frontier orbitals of **1a1** show notable changes, particularly in the Ni–H antibonding characteristics, as the doubly occupied Ni $d_{z^{2}}$ was antibonding with the Fe–H bond in **1a4** and becomes antibonding with the Ni–S in bond in **1a1** (Figure S4). Simultaneously, electron density on the Ni increases as observed by QTAIM (Table 1). NBO analysis reveals that, compared to **1a4**, the Ni orbital coefficient in the ψ_Ni–H–Fe_ orbital of **1a1** shows significant increase of 0.16, with a particularly notable rise in the Ni 3d orbital hybridization coefficient (Figure 2). This indicates that as **1a4** transits to **1a1**, the Ni 3d orbital contribution to the ψ_Ni–H–Fe_ orbital substantially increases. Meanwhile the contributions from the Fe and hydride orbital decrease (Figure 2). Compared to **1a4**, the NBO ψ_Ni–H–Fe_ orbital of **1a1** is more stable by 2.41 eV (Figures 2, S5 and Table 1). The MO and NBO analyses show that the exothermicity and the reduction in hydridic character from **1a4** to **1a1** can be attributed to variations in the stability of the ψ_Ni–H–Fe_ orbital.

**Figure 2:**
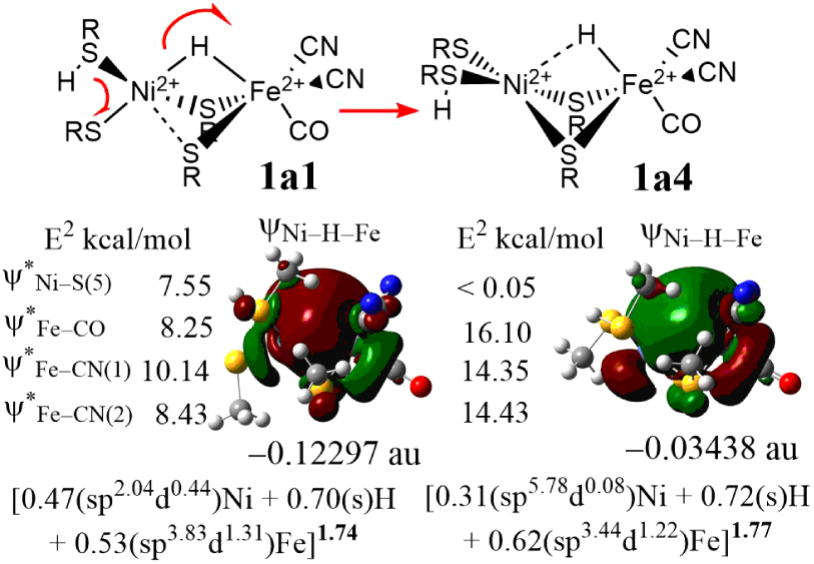
NBO MO (ψ_Ni–H–Fe_), coefficients, hybrids, and occupancy (in bold), and orbital energy (in au) of the [Ni–H–Fe] 3c-2e bond of **1a1** and **1a4**. The stabilization energies *E* ^2^ (in kcal/mol) of the ψ_Ni–H–Fe_ and ψ*_Ni–H–Fe_ with the NBOs of the Fe terminal ligands by second-order perturbation theory are shown in the left side of ψ_Ni–H–Fe_.

By introducing carbon linkers either between two bridging sulfurs as in 2* or between the terminal ligands and bridging sulfurs on the Ni site as in **3,** the biomimetic complexes, 2* and **3**, contain a [Ni­(μ-S_2_)­(μ-H)­Fe] core that resembles **Ni-R** and **1a1**, except that these linkers produce an active site geometry where the hydride is far from the Ni and apical with respect to the square-planar [X_2_NiS_2_] Ni site (2* X = P and **3** X = N). Notably, the structural characteristics of the [Ni­(μ-S_2_)­(μ-H)­Fe] core in these biomimetic complexes closely resemble those of the metastable **1b** (or **1a4**), where the hydride is predominantly bound to the Fe site. To guide synthetic efforts aimed at enhancing the catalytic properties of 2* and **3** for the hydrogen evolution, we examined how the angular geometry of the terminal ligands affects the hydride position and properties of these model complexes, **2** and **3** (Scheme 1). We will present the results for both **2** and **3** first and then compare them to those for **1**.

As anticipated, the M06/DZVP2 approach provided a satisfactory representation of the geometric properties of 2* (Table S5), with a RMSD of 0.299 Å from the experimental structure (Figure S2b). Given that the M06/DZVP2 relative energy (Δ*E*_e_) between **2a** and **2b** (alternative pdt geometries, chair/boat, Figure 1) is only 3.92 kcal/mol (Table S5) and that the energy barrier for the chair/boat flip of the pdt ligand is 9.63 kcal/mol,^67^ **2b** was used for the PES as it would better accommodate variations in the angular geometry between the pdt and the bulky Ni terminal ligands. Thus, the PES scan was conducted by decreasing the θ_X(5)–Ni–S(4)_ angle from 175.9° in **2b** through 165.3° (**2b1**), 145.3° (**2b2**), 125.3° (**2b3**), to 105.3° (**2b4**) (X = P), with the bridging Ni–S(4) and Ni–S(3) bond lengths fixed as observed in **2b** (Figure 1b, Table S4, and Video S3). Among all the conformers of **3**, **3a–3c** and **3d–3f** with alternative linkage geometries, the species **3a** is the most stable species based on the calculated relative energies (Figure S7 and Table S6). Thus, **3a** was used for the PES scan by decreasing the θ_X(5)–Ni–S(4)_ angle from 171.4° (**3a**) through 160.0° (**3a1**), 140.0° (**3a2**), to 120.0° (**3a3**) (X = N) (Figure 1c and Table S6). All on the scans in Figure 1 proceed without an energy barrier; notably, unlike the transition from **1a4** back to **1a1**, the transitions of **2b** to **2b4** and **3a** to **3a3** are endothermic (Figure 1b,c and Table 1). However, like the scan from **1a4** to **1a1**, the NBO [Ni–H–Fe] 3c-2e orbital energies (*E*ψ_Ni–H–Fe_) for the scans **2b** to **2b4** and **3a** to **3a3** decrease by 2.22 and 2.44 eV, respectively. These results suggest that the hydride displacement toward the Ni inherently stabilizes the [Ni­(μ-S_2_)­(μ-H)­Fe] core. The contrasting thermodynamic behaviors observed in the transitions from **2b** to **2b4** and from **3a** to **3a3**, compared to the transition from **1a4** to **1a1**, can be attributed to geometric constraints imposed by the carbon linkers. This highlights the critical role of the carbon linkers in stabilizing the [Ni­(μ-S_2_)­(μ-H)­Fe] core of biomimetic complexes in a geometry different from that observed for **Ni-R**.

In addition to the above trends in the energies, the charges *Q*_X_ (X = H, Ni, Fe), bond lengths, and binding energy of the hydride show related trends (Table 1 and Figure 3). As the hydride shifts closer the Ni (decreasing the θ_X(5)–Ni–S(4)_ angle) from **1a4** to **1a1**, **3a** to **3a3**, and **2b** to **2b4**, the hydride charge (*Q*_H_) progressively increases (Figure 3a), while the Ni–H and Ni–Fe bond lengths (*r*_Ni–H_ and *r*_Ni–Fe_) decrease (Figure 3b). These shifts are accompanied by an increase in stability of the [Ni–H–Fe] 3c-2e bond, which enhances the hydride binding (Figure 3c). These trends lend further evidence that decreasing the θ_X(5)–Ni–S(4)_ angle diminishes the hydridic character while increasing Ni’s nucleophilicity. Thus, in a charge-controlled reaction mechanism, which is primarily influenced by the distribution of charges (electrostatic interactions) on the reactants, the bridging hydride of **1a4**, **3a,** and **2b** possesses higher hydridic character, more favorable for proton bonding via a heterolytic reaction mechanism to generate H_2_. On the other hand, **1a1**, **3a3,** and **2b4** exhibits a greater nucleophilicity on the Ni site, which would favor a homolytic reaction mechanism through a dihydride Ni­(IV) intermediate to generate H_2_.

**Figure 3:**
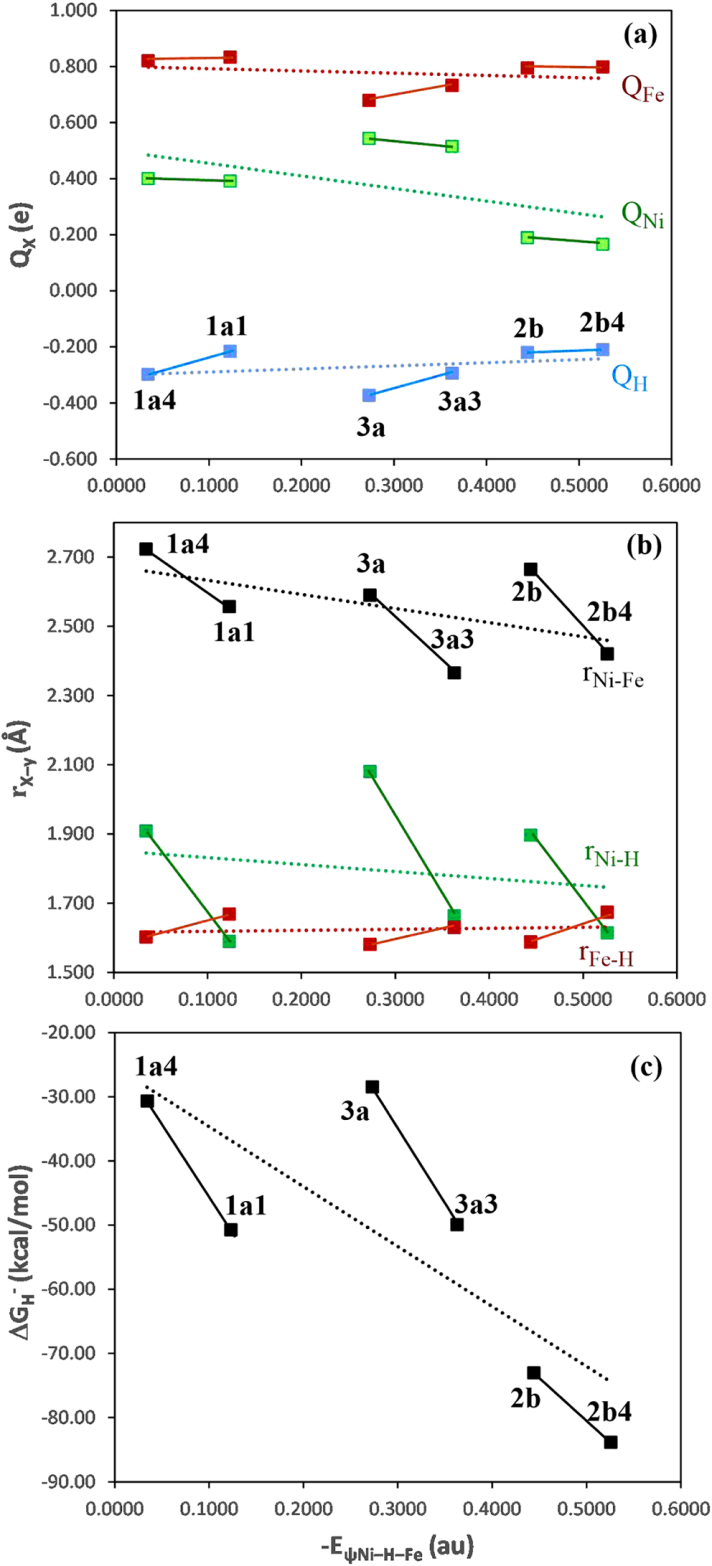
Plot of variations in the electronic and bonding properties as the [Ni–H–Fe] 3c-2e orbital energies (*E*ψ_Ni–H–Fe_) decrease for the **Ni-R** and biomimetic complex models: (a) Charge (Q_X_) on hydride (blue), Ni (green), and Fe (red); (b) Ni–H (green) and Fe–H (red) bond lengths and Ni–Fe distance (black); (c) Hydride (H^–^) binding free energies (Δ*G*_H^–^_) in solution (CH_3_CN). The dashed lines illustrate the overall correlation trends between each property and *E*ψ_Ni–H–Fe_.

Furthermore, as shown in Figure 2, the significant changes in the NBO second-order perturbation energy *E* ^2^ of **1a1**, compared to **1a4**, suggest that the terminal ligands on the Fe and Ni sites can significantly impact the stability of the ψ_Ni–H–Fe_ orbital by the interactions between the unoccupied ψ*_Ni–S(5)_ and ψ*_Fe–CO_/ψ*_Fe–CN_ orbitals and the ψ_Ni–H–Fe_ orbital. Notably, as the [Ni–H–Fe] 3c-2e bonding strengthens from **1a4** to **1a1**, the *E* ^2^ originating from ψ*_Fe–CO_ with σ*_Fe–CO_ antibonding character decreases by 7.85 kcal/mol, leading to a shortening of both the Fe–CO and C≡O bond lengths (Table S4). The significant increase of about 20 cm^–1^ in the calculated C≡O vibrational frequency from **1a4** to **1a1** appears to align well with the substantial shifts in the C≡O vibrational frequency of **Ni-R** observed experimentally.^2^ To evaluate the effects of ligands on the hydridic properties of **Ni-R** and biomimetic complex models, we examined the hydride binding free energies (Δ*G*_H^–^_) in the acetonitrile solution (Table S7). Notably, the calculated Δ*G*_H^–^_ of −73 kcal/mol for **2b** closely matches that for **2a** (−73 kcal/mol) and the experimental value for 2a* (−79 kcal/mol).^33^ Overall, the Δ*G*_H^–^_ of these models appear to correlate with the *E*ψ_Ni–H–Fe_, as a function of the θ_X(5)–Ni–S(4)_ angle, both deceasing in the order of **1a4** > **1a1**, **3a** > **3a3**, and **2b** > **2b4** (Table 1 and Figure 3c). Interestingly, like the θ_X(5)–Ni–S(4)_ angle, the *E*ψ_Ni–H–Fe_ values decrease in the order: **1a4** > **3a** > **2b**, and **1a1** > **3a3** > **2b4**, but the Δ*G*_H^–^_ shows: **1a4** ≈ **3a** > **2b**, and **1a1** ≈ **3a3** > **2b4**. Thus, **1** (or **Ni-R**) and **3** are predicted to have similar but higher hydride activity than 2 (or 2*). Our results strongly suggest that the hydride activity decreases as the hydride shifts toward the Ni across the transitions from **1a4**, **2b**, and **3a** to **1a1**, **3a3**, and **2b4**, respectively (Figures 3 and S8). As mentioned above, both experimental and theoretical studies support that displacing hydride toward Ni facilitates H_2_ formation via either heterolytic or homolytic reaction pathways.^6,11^ However, the higher Δ*G*_H^–^_ and lower *Q*_H_ (*vide supra*) observed for **1a1**, **2b4**, and **3a3** compared to their precursors presents a challenge to fully understanding the activity of [Ni­(μ-S_2_)­(μ-H)­Fe] core in **Ni-R** versus biomimetic complexes. Thus, additional factors may help facilitate H_2_ formation in **Ni-R**. It is worth noting that as the strength of [Ni–H–Fe] 3c-2e bonding increases, the frontier orbitals, specifically the doubly occupied Ni $d_{z^{2}}$ and the unoccupied Ni $d_{x^{2}-y^{2}}$ , move closer in energy, leading to substantial changes in the hydride character within these orbitals (Figures S3 and S4). This increased hydride contribution in the frontier orbitals may significantly enhance H_2_ formation via an orbital-controlled reaction mechanism that lies intermediate between a homolytic and heterolytic pathways. Mimicking the [Ni­(μ-S_2_)­(μ-H)­Fe] core structure is a crucial step for the rational design of biomimetic catalyst, as this will provide a full understanding of how the structure of the [Ni­(μ-S_2_)­(μ-H)­Fe] core contributes to the H_2_ formation.

Our findings demonstrated that the carbon linkers in reported biomimetic complexes are critical in maintaining the [Ni­(μ-S_2_)­(μ-H)­Fe] core structure, likewise the protein backbones stabilize the **Ni-R** active site. This insight suggests a strategy for the rational design of biomimetic complexes with higher activity might involve adjusting the θ_X(5)–Ni–S(4)_ angle (X = P, N) of 2a* and **3** by modifying the carbon linkers between the Ni terminal ligands and bridging thiolate ligands. Changing this angle might lead the complexes to more closely mimic the **Ni-R** active site in enzymes. Among a series of the “designer” complexes **4**–**10** (Scheme S1), **4**–**8** and **9a** exhibited stronger resemblance to the model complex **1a1** of the **Ni-R** active site in terms of hydride position and the electronic properties of the [Ni­(μ-H)­(μ-S)­2Fe] core (Figures 4 and S9).

**Figure 4:**
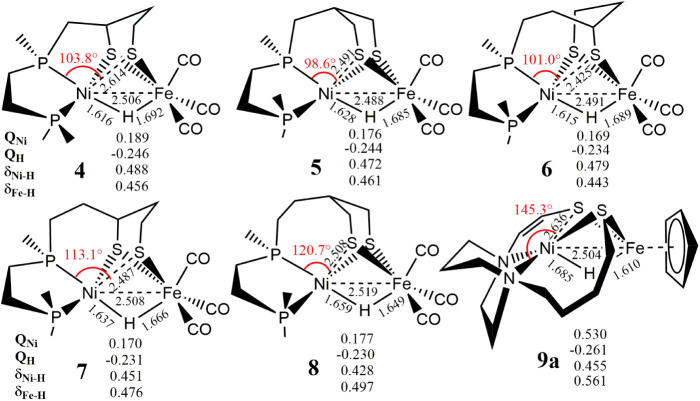
Optimized geometric parameters (θ_X(5)–Ni–S(4)_ in deg and *r*_M–H_ in Å) and QTAIM topological properties (charge *Q* and electron delocalization index δ in au) of the designed biomimetic complexes **4**–**8** (X = P) and **9a** (X = N).

The calculated Ni–Fe distance, Ni–H, and Fe–H bond lengths in these complexes ranged from 2.488 to 2.519 Å, 1.615 to 1.685 Å, and 1.610 to 1.692 Å, respectively (Figures 4, S9 and Table S8). Especially, the biomimetic complex **2a** appears to exhibit a higher degree of adaptability in achieving species **4**–**8** that feature a [Ni­(μ-H)­(μ-S)_2_Fe] structural motif like that in **Ni-R**, as there appear to be more alternative options for modifying the carbon linker. The virtual complexes **4**–**8** exhibited total electron delocalization indices for the Ni–H and Fe–H bonds (δ_Ni–H–Fe_) ranging from 0.925 to 1.016 au, which are comparable to the value of 0.952 au calculated for **1a1**. Notably, compared to **1a1**, the smaller positive charge on the Ni (*Q*_Ni_) and the larger negative charger on the hydride (*Q*_H_) in **4**–**8** suggest that these virtual complexes might possess not only a higher nucleophilic Ni site, enabling an oxidative addition reaction for H_2_ evolution via a homolytic pathway, but also exhibit a more nucleophilic hydride, facilitating the H_2_ evolution via a heterolytic pathway. Additionally, the virtual complexes **9a** and **9b**, characterized by a more nucleophilic hydride (*Q*_H_) and a longer bridging Ni–S(4) distance of 2.64–2.80 Å compared to **1a1** may offer a more favorable proton transfer channel through the bridging S(4) for H_2_ formation via a heterolytic reaction pathway compared to their native complex **3**. It is noteworthy that a recent comprehensive DFT study on H_2_ evolution catalyzed by a [Ni­(N2S2)·Fe­(CO)­Cp]^+^ complex revealed a significantly lower energy pathway through an E­[ECEC] electrochemical reaction mechanism (E = electron transfer, C = chemical reaction, here corresponding to a proton transfer).^45,46^ In this mechanism, the [(N2S2)­Ni­(μ-H)­Fe­(CO)­Cp]^+^ intermediate undergoes a reduction to form a Ni­(I) site, followed by protonation on a bridging sulfur, leading to H_2_ formation via a heterolytic reaction pathway. Overall, from the perspective of charge-controlled reactions, in comparison to their native complexes, 2a* and **3**, the virtual biomimetic complexes, **4**–**9**, should demonstrate significant improvement in their reactivity for H_2_ formation.

## Conclusions

Our CCSD and DFT calculations indicate that the equilibrium geometry of the [X_2_Ni­(μ-S)_2_(μ-H)­Fe] core (X = S, P, N) differs in **Ni-R** and the synthetic biomimetic complexes (2* and **3**). While **Ni-R** inherently favors a square-planar [X_2_NiSH] geometry with an apically positioned thiolate, the biomimetic complexes favor a square-planar [X_2_NiS_2_] geometry with an apically positioned hydride that is bonded more strongly to Fe. Moving a bridging thiolate to the apical site, as in **Ni-R**, shifts the hydride from the Fe to a basal bonding site on Ni. This displacement of the bridging hydride from the Fe site to the Ni site is facilitated by [Ni–H–Fe] 3c-2e bonding, which is only possible when the hydride is in the basal Ni site. Model complexes mimicking the geometry of **Ni-R** can be constructed by manipulating the geometric torsion of the Ni terminal ligands relative to the bridging ligands. Specifically, adjusting the θ_X(5)–Ni–S(4)_ angle (X = P, N) through modifications of the carbon linkers between the Ni terminal ligands and bridging thiolate ligands of 2* and **3**, generates a series of virtual complexes that achieve a similar [Ni­(μ-S_2_)­(μ-H)­Fe] core with structural and electronic properties similar to those found in **Ni-R**. Finally, compared to the **Ni-R** model complex **1a1**, the modified biomimetic complexes **4**–**8** based on **2** exhibit improved protonation capabilities at either the Ni or the bridging hydride, while the complex **9** by modifying **3** demonstrates enhanced nucleophilicity on the bridging hydride, facilitating H_2_ formation. These findings provide valuable guidance for the rational design and modifications of biomimetic complexes that may more closely mimic the biological functions of **Ni-R**. Further systematic studies are underway to elucidate the reasonable enzymatic mechanisms of **Ni-R** and identify key factors that activate hydride. Leveraging high-quality descriptors derived from the rate-determining step of a well-defined enzymatic process, we aim to design and optimize biomimetic complexes with improved catalytic efficiency for H_2_ formation.

## Supplementary Material











## Notes

Computational details; Scheme S1, Figures S1–S9, Tables S1–S8 (PDF)
Cartesian coordinates of calculated models at the M06/DZVP2 level (PDF)
Visualizing structural transitions of **1b** → **1a** → **1b** (MP4)
Visualizing structural transitions of **1b** → **1a1** → **1a** → **1a1** → **1b** (MP4)
Visualizing structural transitions of **2a** → **2b** → **2b4** → **2b** → **2a** (MP4)
