# Supplementary material for: How Geometric Constraints Control the Hydride Position and Activity in [NiFe]-Hydrogenases and Their Biomimetic Complexes
Source: Inorg Chem. 2025 May 9;64(20):10078–86. doi: 10.1021/acs.inorgchem.5c00670 (PMC12117559; doi:10.1021/acs.inorgchem.5c00670)
Supplement: Supplementary file 1 [file ic5c00670_si_001.pdf]

Supporting Information I

How Geometric Constraints Control the Hydride Position and Activity in [NiFe]-Hydrogenases and Their Biomimetic Complexes

Shuqiang Niu* and Michael B. Hall*

Department of Chemistry, Texas A&M University, College Station, TX 77843-3257

*To whom correspondence may be addressed. E-mail: sn72@tamu.edu and MBHall@tamu.edu

I. Computational Details

The full and constraint geometry optimizations of the model complexes **1** of the **Ni-R** active site were performed using coupled-cluster singles-and-doubles (CCSD) and density functional theory (DFT) methods. In the CCSD calculations, all valence electrons were correlated, including the Ni and Fe 3s and 3p electrons. The 6-31G** basis sets were employed for the Ni, Fe, S, and hydrogens involved in the H_2 formation/cleavage, while the STO-3G basis sets were utilized for methyl, CO, and CN groups. Spin-unrestricted DFT calculations with extra fine grids for numerical integration (target accuracy of 10^{-8} au in energy calculations) were performed. Five functionals,[1,2] BP86, TPSS, B3LYP, M06, and LC-ωBPEh, with two basis sets, DZVP2 and Def2-TZVP, were assessed for their accuracy in determining geometric and energetic properties of **Ni-R** active site models, **1a**, **1a1**, and **1b** (Table S2-S3).

The full optimizations of **1a** using various density functionals in the gas phase typically result in a structure with a planar square [$S_{(5)}S_{(6)}NiS_{(3)}H$] core and a long Ni–S4 distance ranging from 2.69 to 3.87 Å (Table S2). Notably, DFT constraint optimizations of **1a1** with the fixed bridging Ni–S4 distance matching the experimental value of 2.54 Å observed in **Ni-R** led to a very good description of the geometry parameters of the active site, surpassing even the results of the CCSD calculations (Table S3). Moreover, the constraint optimization of **1a1** at the M06/DZVP2 level of theory, maintaining the fixed bridging Ni–S4 distance at the experimental value of 2.54 Å observed in **Ni-R**, provided a very good description of the geometry parameters with the RMSD values of 0.250 and

0.299 Å for the **Ni-R** active site and biomimetic complex **2a***, respectively, (Figure S1, Table S3, and Table S5). Furthermore, To evaluate an electronic effects of protein environments (ε ≈ 4.0) on the bonding structure of the **Ni-R** active site, the full geometry optimizations were carried out on a conversion process of **1a** to **1b** in the diethylamine solution phase (ε = 3.6) utilizing the SMD (Solvation Model Density) continuum solvation model.[3]

Previous studies have demonstrated the effiectiveness of the M06/DZVP2 approach in accurately describing geometries, inner-sphere reduction energies, thermodynamic and kinetic properties of redox and catalytic sites in various metalloenzymes like bimetallic hydrogenases,[4] iron-sulfur electron transfer proteins,{Niu, 2011 #4296} and blue-copper proteins.[5] In our benchmark study for this work, we further assessed the fourteen density funtionals with the nine basis sets commonly used in DFT calculations for transition metal systems. By comparing the experimental structures[6,7] and photoelectron spectroscopy data[8] of $[M(mnt)_2]^{n-}$ (M = Ni, Fe; mnt = 1,2-$S_2C_2(CN)^{2-}$; n = 2, 1), we confirmed that the M06/DZVP2 (or M06/DGDZVP2) provided a reliable balance of computational cost and accuracy for describing geometries and energies of both Ni- and Fe-bis(dithiolene) complexes (Figure S1). Therefore, M06/DZVP2 was primarily used for geometry optimizations and energy calculations in this study.

The potential energy surface (PES) scan at the M06/DZVP2 level involved increasing the angle along $\theta_{S(5)-Ni-S(4)}$ from 115° (**1a1**) to 140° (**1a2**), 160° (**1a3**) and finally 180° (**1a4**), while fixing the bridging Ni–S(4) and Ni–S(3) distances at 2.54 and 2.21 Å, respectively, as observed in **Ni-R** and **1a1**. The **2a** $[(CO)_3Fe(pdt)(\mu\text{-}H)Ni(dmpe)]^+$ (dmpe = 1,2-$C_2H_4(PMe_2)_2$) is a simplified model of a biomimetic complex **2a*** $[(CO)_3Fe(pdt)(\mu\text{-}H)Ni(dppe)]^+$. Again, the full optimization of **2a*** and **2a** at the M06/DZVP2 level showed very good agreement with the experimental structure of **2a*** (Figure S2, Table S5). The **2b** is a pdt flipping-isomer of **2a**, which is less stable by 3.92 kcal/mol in the total electron energy at the M06/DZVP2 level than **2a** but may be beneficial to a torsion of the dmpe ligand. In the PES scan from **2b** through **2b1-2b3** to **2b4**, with the variations in the $\theta_{P(5)-Ni-S(4)}$ angle (Figure S6), the bridging Ni–S(4) and Ni–S(3) bonds were fixed to the

calculated values of **2b**. Regarding the proposed biomimetic model **3**, there are two sets of conformational isomers of **3a**-**3c** and **3d**-**3f** (Figure S7). Since **3a** is most stable species and allows for greater torsion of the [N2S2] ligand than the symmetric isomers, the **3a** was selected to probe the effects of the [N2S2] ligand on the hydride position in this study.

The relative energies (ΔE_e) and the relative free energy (ΔG) were determined using the total electronic energy (E_e) and Gibbs free energy (G) of molecules, calculated at either fully optimized or constrained optimized geometries. Gibbs free energies ($G = H–TS$) at temperature of 298.15 K were obtained by summing the calculated E_e and thermal energy correction to G. The thermal energy correction to enthalpy ($H_{corr} = ZPE+E_{vib}+E_{rot}+E_{rot}+RT$) and the total entropy (S) were derived from a frequency calculation. Additionally, the hydride bonding energies (ΔG_{H^-}) of the complexes were calculated using the M06/DZVP2 method and are defined as:

$$\Delta G_{H^-} = G(MH^-) – [G(M) + G(H^-)]$$

The calculated ΔG_{H^-} of -73.03 kcal/mol in MeCN solutions for **2b** closely matches that for **2a** (−72.50 kcal/mol) and the experimental value for **2a*** (−79 kcal/mole) (Table S6).[9] The quantum theory of atoms in molecules (QTAIM) analysis[10] and natural bond orbital (NBO)[11] were employed to evaluate the atomic charges on the atom A (Q_A), the electron delocalization indexes of an A−B bond ($\delta_{A–B}$), the orbital occupancy $Q_{Ni–H–Fe}$, orbital energy $E\psi_{Ni–H–Fe}$, and NBO stabilization energies E^2 of donor-acceptor interactions (using 2nd-order perturbation theory).

All DFT calculations were conducted using the NWChem software packages.[12] The G09 computational chemistry software package[13] was utilized for CCSD and NBO calculations while the QTAIM calculations were performed with the AIMALL software package.[14] Structural and electronic structural visualizations and analysis were carried out using the Ampac Graphical User Interface (*AGUI*),[15] Gauss View,[13] ChemOffice,[16] Molden,[17] and Chimrea.[18] The simulated reaction pathways were visualized and converted into videos using *AGUI* or GaussView, with the IRC was generated by our mLST-IRC program. The mLST-IRC program allows for the generation of reaction trajectories by

linking key points such as reactants, intermediates, transition states, and products through multi-linear synchronous transit (mLST) calculations.

References

(1) Cramer, C. J.; Truhlar, D. G. *Phys Chem Chem Phys* **2009**, *11*, 10757.
(2) Cramer, C. J. *Essentials of computational chemistry : theories and models*; 2nd ed.; John Wiley & Sons Inc., 2004.
(3) Marenich, A. V.; Cramer, C. J.; Truhlar, D. G. *J. Phys. Chem. B* **2009**, *113*, 6378.
(4) Niu, S. Q.; Nelson, A. E.; De La Torre, P.; Li, H.; Works, C. F.; Hall, M. B. *Inorg. Chem.* **2019**, *58*, 13737.
(5) Niu, S. Q.; Huang, D. L.; Dau, P. D.; Liu, H. T.; Wang, L. S.; Ichiye, T. *J. Chem. Theory Comput.* **2014**, *10*, 1283.
(6) Gama, V.; Henriques, R. T.; Bonfait, G.; Pereira, L. C.; Waerenborgh, J. C.; Santos, I. C.; Duarte, M. T.; Cabral, J. M. P.; Almeida, M. *Inorg. Chem.* **1992**, *31*, 2598.
(7) Silva, R. A. L.; Neves, A. I. S.; Lopes, E. B.; Santos, I. C.; Coutinho, J. T.; Pereira, L. C. J.; Rovira, C.; Almeida, M.; Belo, D. *Inorg. Chem.* **2013**, *52*, 5300.
(8) Waters, T.; Wang, X. B.; Woo, H. K.; Wang, L. S. *Inorg. Chem.* **2006**, *45*, 5841.
(9) Barton, B. E.; Rauchfuss, T. B. *J. Am. Chem. Soc.* **2010**, *132*, 14877.
(10) Corts-Guzman, F.; Bader, R. F. W. *Coord. Chem. Rev.* **2005**, *249*, 633.
(11) Weinhold, F.; Landis, C. R. *Discovering Chemistry With Natural Bond Orbitals*; John Wiley & Sons Inc.: Hoboken, New Jersey, 2012.
(12) Aprà, E.; Bylaska, E. J.; Jong, W. A. d.; Govind, N.; Kowalski, K.; Straatsma, T. P.; Valiev, M.; Dam, H. J. J. v.; Alexeev, Y.; Anchell, J.; Anisimov, V.; Aquino, F. W.; Atta-Fynn, R.; Autschbach, J.; Bauman, N. P.; Becca, J. C.; Bernholdt, D. E.; Bhaskaran-Nair, K.; Bogatko, S.; Borowski, P.; Boschen, J.; Brabec, J.; Bruner, A.; Cauët, E.; Chen, Y.; Chuev, G. N.; Cramer, C. J.; Daily, J.; Deegan, M. J. O.; DunningJr., T. H.; Dupuis, M.; Dyall, K. G.; Fann, G. I.; Fischer, S. A.; Fonari, A.; Früchtl, H.; Gagliardi, L.; Garza, J.; Gawande, N.; Ghosh, S.; Glaesemann, K.; Götz, A. W.; Hammond, J.; Helms, V.; Hermes, E. D.; Hirao, K.; Hirata, S.; Jacquelin, M.; Jensen, L.; Johnson, B. G.; Jónsson, H.; Kendall, R. A.; Klemm, M.; Kobayashi, R.; Konkov, V.; Krishnamoorthy, S.; Krishnan, M.; Lin, Z.; Lins, R. D.; Littlefield, R. J.; Logsdail, A. J.; Lopata, K.; Ma, W.; Marenich, A. V.; Campo, J. M. d.; Mejia-Rodriguez, D.; Moore, J. E.; Mullin, J. M.; Nakajima, T.; Nascimento, D. R.; Nichols, J. A.; Nichols, P. J.; Nieplocha, J.; Otero-de-la-Roza, A.; Palmer, B.; Panyala, A.; Pirojsirikul, T.; Peng, B.; Peverati, R.; Pittner, J.; Pollack, L.; Richard, R. M.; Sadayappan, P.; Schatz, G. C.; Shelton, W. A.; Silverstein, D. W.; Smith, D. M. A.; Soares, T. A.; Song, D.; Swart, M.; Taylor, H. L.; Thomas, G. S.; Tipparaju, V.; Truhlar, D. G.; Tsemekhman, K.; Voorhis, T. V.; Vázquez-Mayagoitia, Á.; Verma, P.; Villa, O.; Vishnu, A. *J. of Chem. Phys.* **2020**, *152*, 184102.
(13) Frisch, M. J. T., G. W.; Schlegel, H. B.; Scuseria, G. E.; Robb, M. A.; Cheeseman, J. R.; Scalmani, G.; Barone, V.; Petersson, G. A.; Nakatsuji, H.; Li, X.; Caricato, M.; Marenich, A. V.; Bloino, J.; Janesko, B. G.; Gomperts, R.; Mennucci, B.; Hratchian, H. P.; Ortiz, J. V.; Izmaylov, A. F.; Sonnenberg, J. L.; Williams-Young, D.; Ding, F.; Lipparini, F.; Egidi, F.; Goings, J.; Peng, B.; Petrone, A.; Henderson, T.;

Ranasinghe, D.; Zakrzewski, V. G.; Gao, J.; Rega, N.; Zheng, G.; Liang, W.; Hada, M.; Ehara, M.; Toyota, K.; Fukuda, R.; Hasegawa, J.; Ishida, M.; Nakajima, T.; Honda, Y.; Kitao, O.; Nakai, H.; Vreven, T.; Throssell, K.; Montgomery, J. A., Jr.; Peralta, J. E.; Ogliaro, F.; Bearpark, M. J.; Heyd, J. J.; Brothers, E. N.; Kudin, K. N.; Staroverov, V. N.; Keith, T. A.; Kobayashi, R.; Normand, J.; Raghavachari, K.; Rendell, A. P.; Burant, J. C.; Iyengar, S. S.; Tomasi, J.; Cossi, M.; Millam, J. M.; Klene, M.; Adamo, C.; Cammi, R.; Ochterski, J. W.; Martin, R. L.; Morokuma, K.; Farkas, O.; Foresman, J. B.; Fox, D. J. *Gaussian 09, Revision A.01*; Gaussian, Inc.: Wallingford CT, 2009.
(14) Keith, T. A. In *(Version 19.10.12)*; TK Gristmill Software: Overland Park KS, USA, 2019.
(15) Glaser, R. *J. Am. Chem. Soc.* **2009**, *131*, 13564.
(16) Buntrock, R. E. *J. Chem. Inf. Comput. Sci.* **2002**, *42*, 1505.
(17) Schaftenaar, G.; Noordik, J. H. *J. Comput.-Aided Mol. Des.* **2000**, *14*, 123.
(18) Pettersen, E. F.; Goddard, T. D.; Huang, C. C.; Couch, G. S.; Greenblatt, D. M.; Meng, E. C.; Ferrin, T. E. *J. Comput. Chem.* **2004**, *25*, 1605.
(19) Li, H. X.; Niu, S. Q.; Hall, M. B. *Unpublished work.*
(20) Ogata, H.; Nishikawa, K.; Lubitz, W. *Nature* **2015**, *520*, 571.
(21) Barton, B. E.; Whaley, C. M.; Rauchfuss, T. B.; Gray, D. L. *J. Am. Chem. Soc.* **2009**, *131*, 6942.
(22) Huynh, M. T.; Schilter, D.; Hammes-Schiffer, S.; Rauchfuss, T. B. *J. Am. Chem. Soc.* **2014**, *136*, 12385.
(23) Schilter, D.; Camara, J. M.; Huynh, M. T.; Hammes-Schiffer, S.; Rauchfuss, T. B. *Chem. Rev.* **2016**, *116*, 8693.
(24) Carroll, M. E.; Barton, B. E.; Gray, D. L.; Mack, A. E.; Rauchfuss, T. B. *Inorg. Chem.* **2011**, *50*, 9554.
(25) Zhang, F.; Woods, T. J.; Rauchfuss, T. B. *Organometallics* **2023**, *42*, 1607.
(26) Basu, D.; Bailey, T. S.; Lalaoui, N.; Richers, C. P.; Woods, T. J.; Rauchfuss, T. B.; Arrigoni, F.; Zampella, G. *Inorg. Chem.* **2019**, *58*, 2430.
(27) Ogo, S.; Ichikawa, K.; Kishima, T.; Matsumoto, T.; Nakai, H.; Kusaka, K.; Ohhara, T. *Science* **2013**, *339*, 682.
(28) Ogo, S.; Kishima, T.; Yatabe, T.; Miyazawa, K.; Yamasaki, R.; Matsumoto, T.; Ando, T.; Kikkawa, M.; Isegawa, M.; Yoon, K. S.; Hayami, S. *Science Advances* **2020**, *6*, eaaz8181.

II. Scheme, Figure, Video, and Table

Scheme S1.

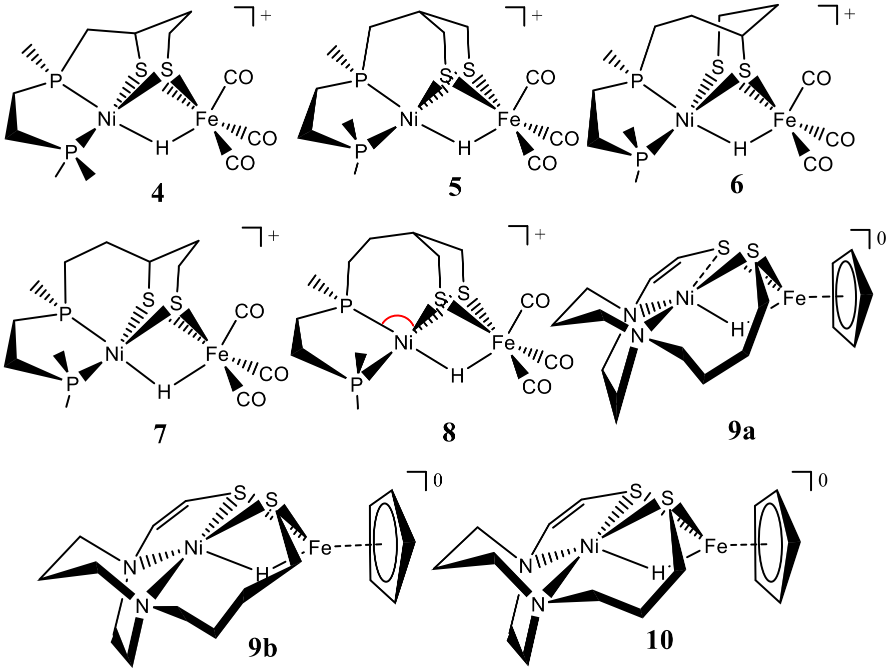

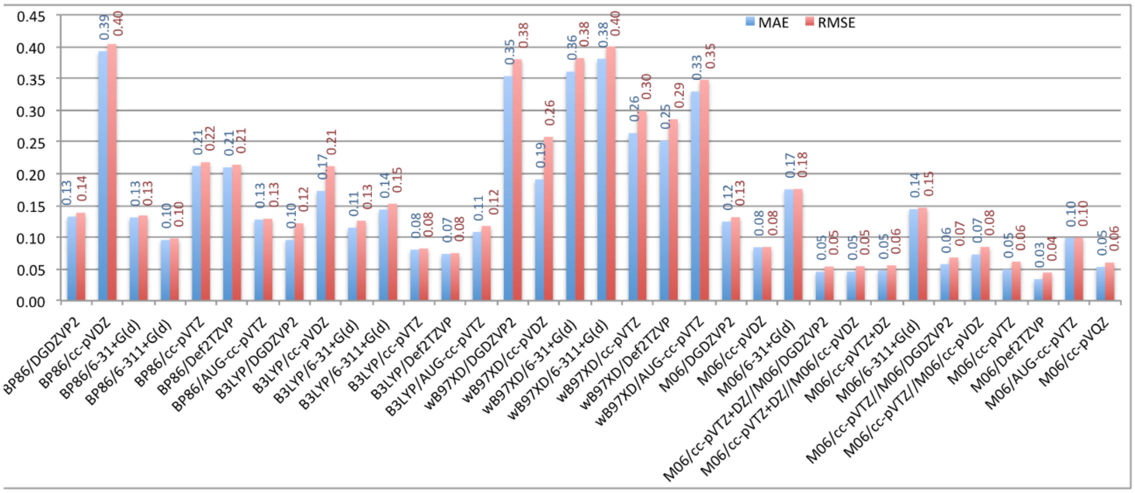

Figure S1. Mean absolute error and root-mean-square error (MAE and RMSE, respectively, in eV) for the calculated results against the experimental adiabatic and vertical detachment energies (ADE and VDE, respectively, in eV) of $[Ni(mnt)_2]^{2-/1-}$ complexe[8] by using nine basis sets together with BP86, B3LYP, ωB97XD, and M06 functionals.[19]

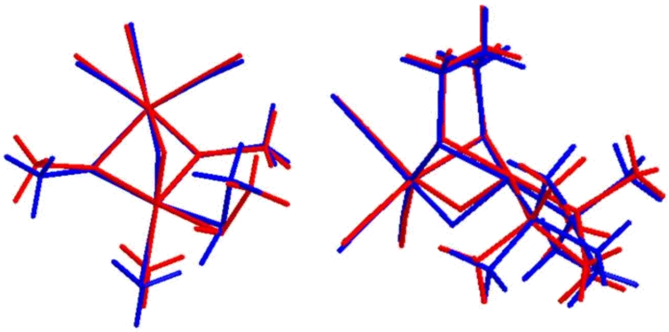

(a) (b)

Figure S2. Geometry overlay of the optimized **1a1** and **2a** (blue) with the experimental core structure (red) of the **Ni-R** active site (a) and **2*** (b), respectively. The root-mean-square deviations (RMSD) without proton atoms are 0.250 and 0.299 Å,[18] respectively.

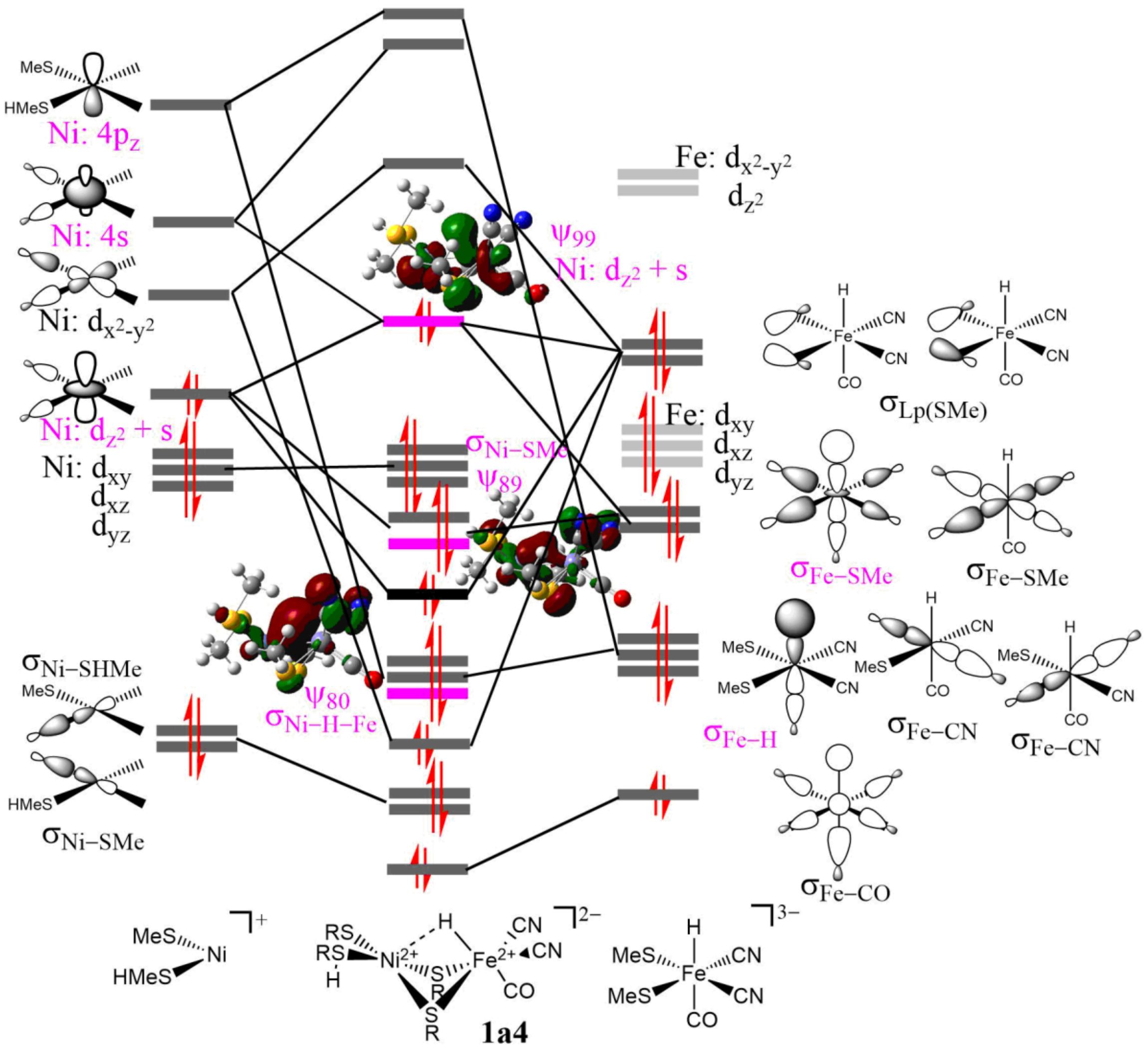

Figure S3. Schematic the MO interaction diagram between an octahedral $[FeH(SMe)_2(CN)_2(CO)]^{3-}$ and a right triangle $[Ni(SMe)(SMeH)]^{+}$ fragments for **1a4**. The DFT MOs and levels of the 3c-2e bonding orbital $\psi_{Ni\text{-}H\text{-}Fe}$ (ψ_{80}), $\sigma_{Ni\text{–}SMe}$, and Ni $3d_{z^2}$ are displayed in magenta. The Fe 3d orbitals which are only weakly involved in the interaction with the Ni site are displayed in light gray to highlight key interactions at the Ni site, displayed in dark grey. The original coordinate systems of the Ni and Fe fragments are retained to clearly distinguish the atomic orbital types during MO analysis.

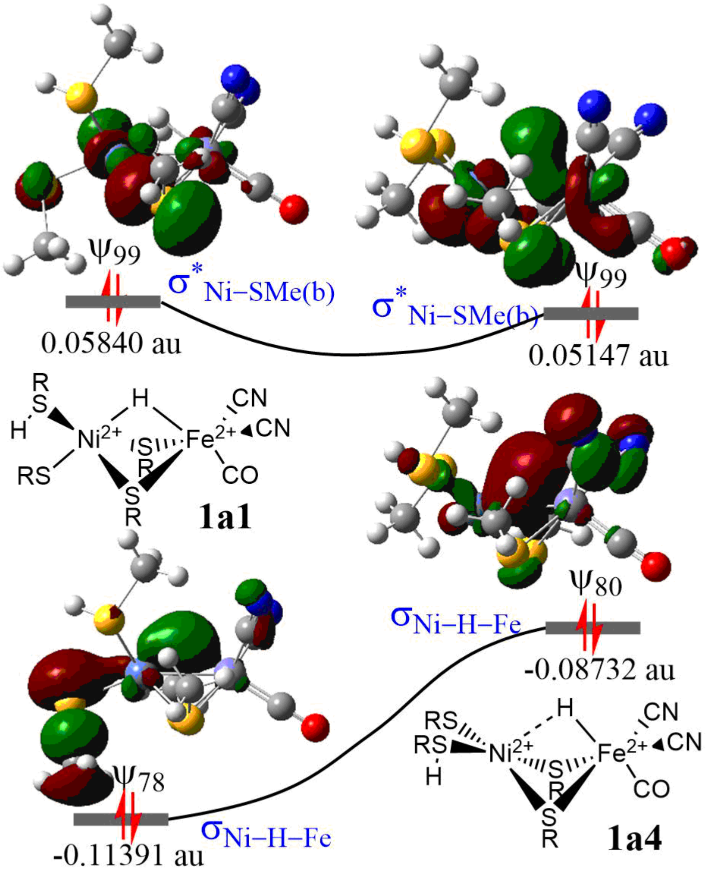

Figure S4. The [Ni-H-Fe] 3c-2e bonding MOs, Ni $3d_{z^2}$ type MOs, and their energies of **1a1** (ψ_{78}, ψ_{99}) and **1a4** (ψ_{80}, ψ_{99}).

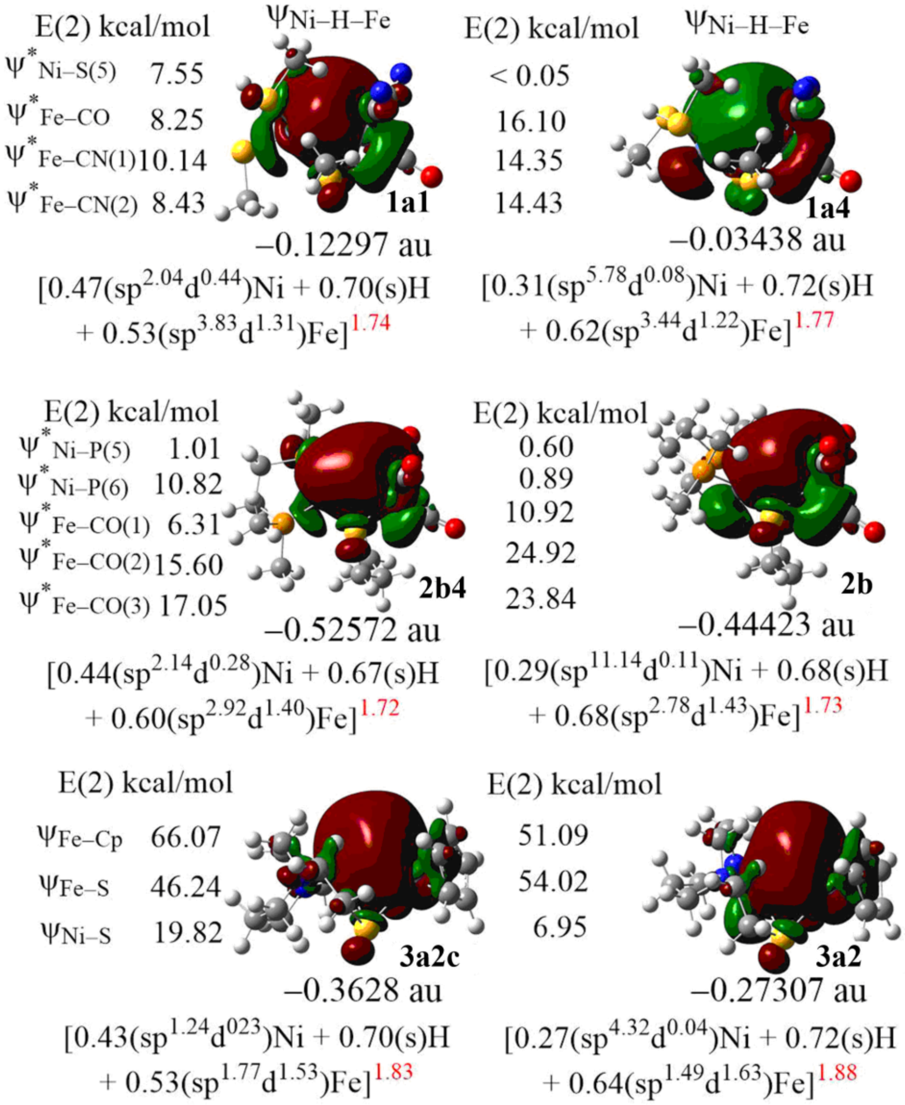

Figure S5. The NBO MO ($\psi_{Ni\text{-}H\text{-}Fe}$), coefficients, hybrids, and occupancy (red), and orbital energy (in au) of the [Ni-H-Fe] 3c-2e bond of **1a1**, **2b**, **3a2**, and their conformers. The stabilization energies E(2) (in kcal/mol) of the $\psi_{Ni\text{-}H\text{-}Fe}$ and $\psi^*_{Ni\text{-}H\text{-}Fe}$ with the NBOs of the Fe terminal ligands by 2nd-order perturbation theory are shown in the left side of $\psi_{Ni\text{-}H\text{-}Fe}$.

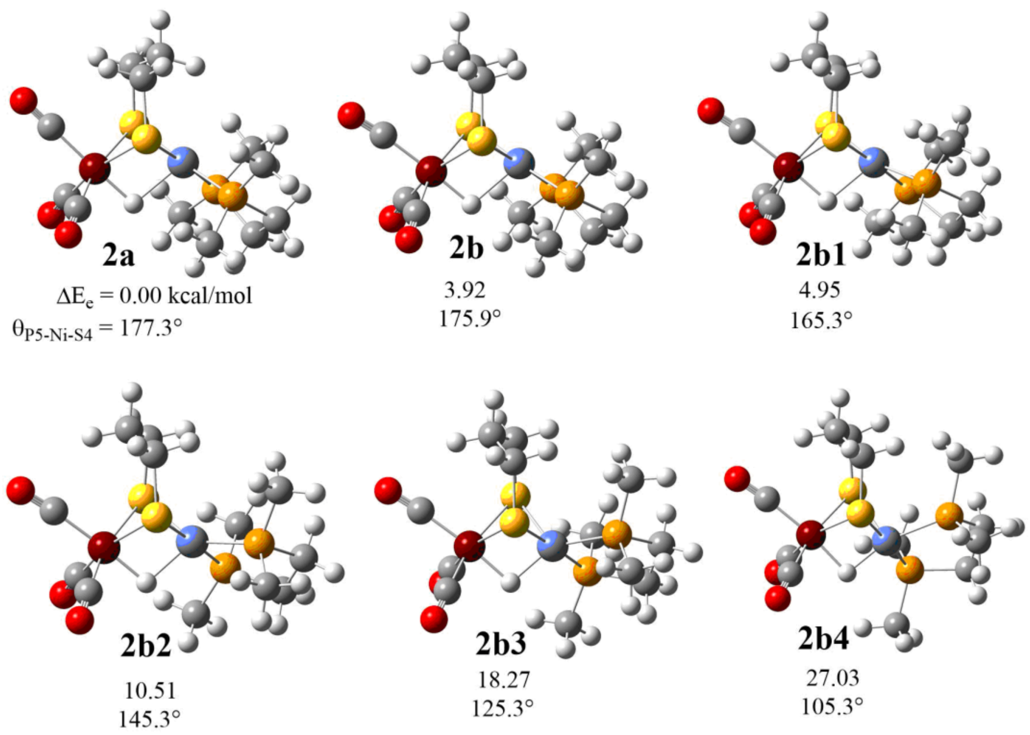

Figure S6. The optimized geometries and relative total electron energy (ΔE_e in kcal/mol) of the complex isomers **2a** and **2b**. In the constrained geometry optimizations of **2b1**-**2b4** for the PES scanning, the $\theta_{P(5)–Ni–S(4)}$ angle and the bridging Ni–S(4) and Ni–S(3) bonds were fixed as observed in **2b**.

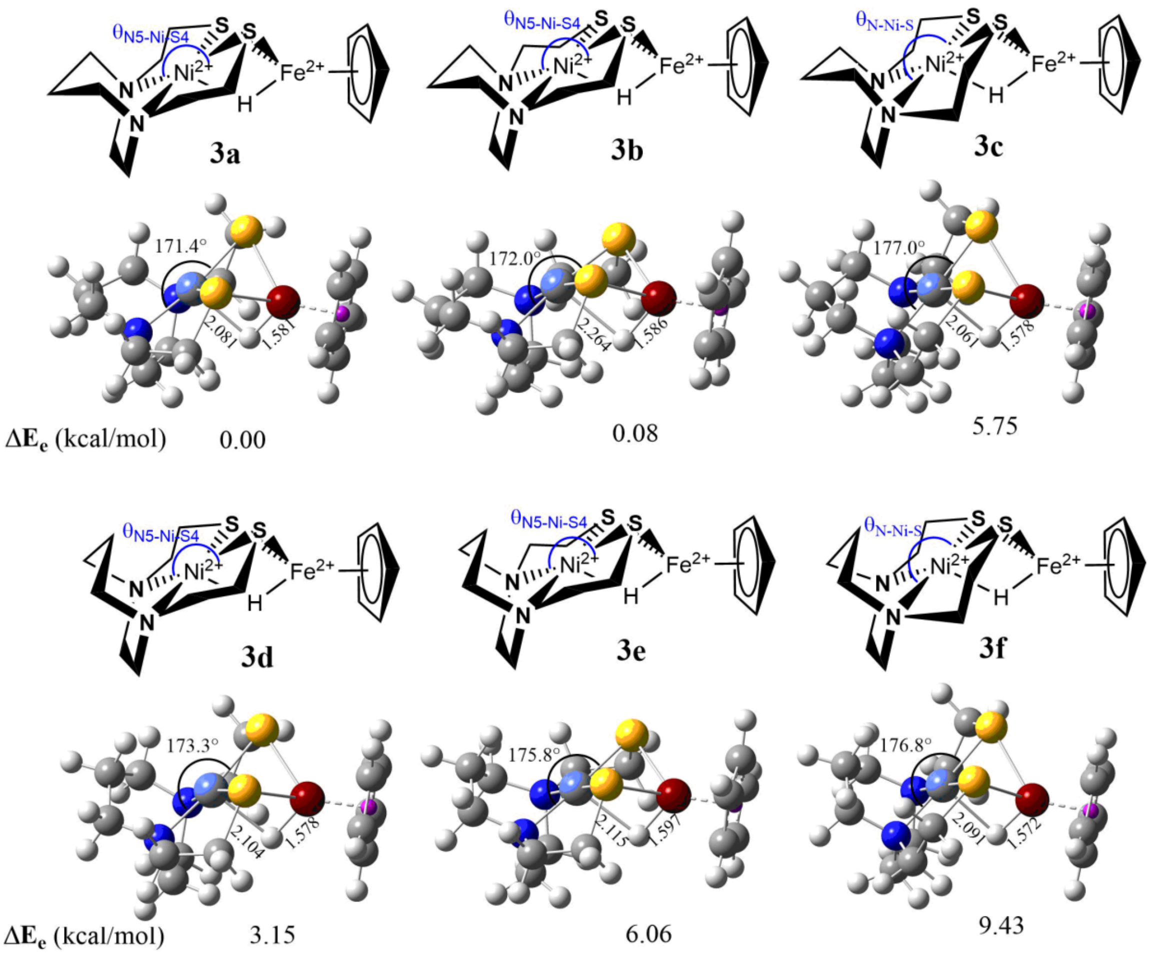

Figure S7. The optimized geometric parameters ($\theta_{N5-Ni-S4}$ in ˚ and r_{M-H} in Å) and relative total electron energy (ΔE_e in kcal/mol) of the isomers of biomimetic complexes **3a**-**c** and **3d**-**f**.

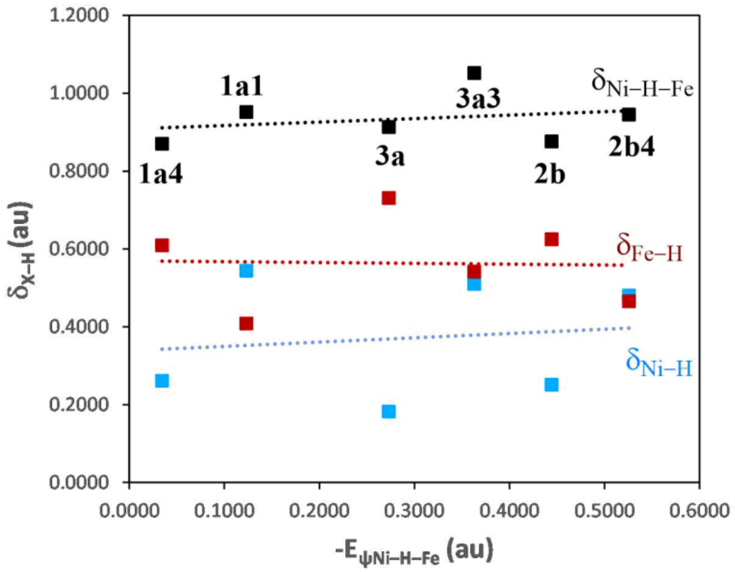

Figure S8. The plot of variations in electron delocalization index ($\delta_{X–H}$) of the Ni–H (blue), Fe–H (red), and Ni–H–Fe (black) bonds as the [Ni–H–Fe] 3c-2e orbital energies ($E\psi_{Ni\text{-}H\text{-}Fe}$) decrease for the Ni-R and biomimetic complex models.

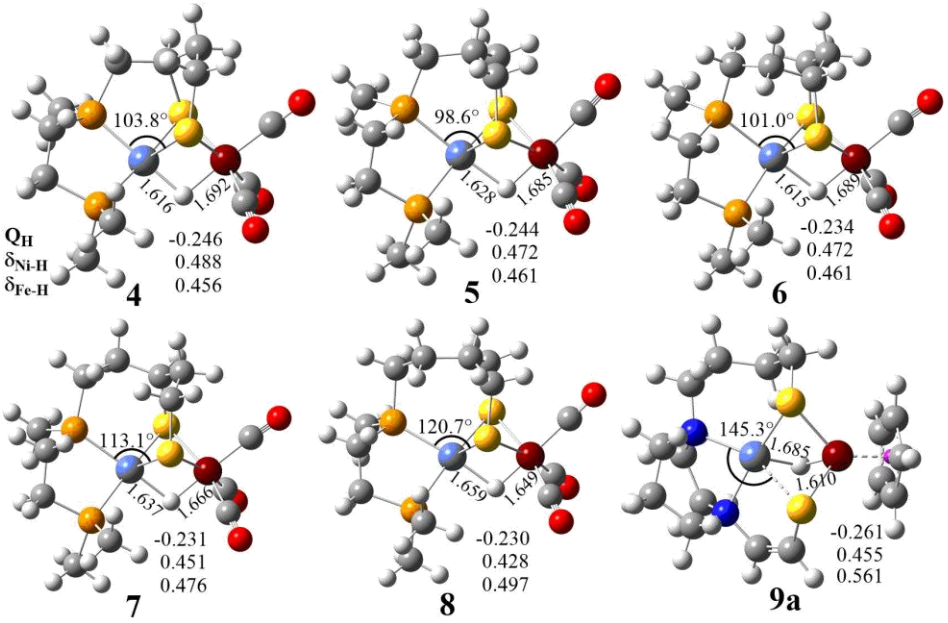

Figure S9. The optimized 3D structures, geometric parameters ($\theta_{X5-Ni-S4}$ in ° and r_{M-H} in Å) and QTAIM topological properties (charge Q and electron delocalization index δ in au) of the designed biomimetic complexes **4–8** (X = P) and **9a** (X = N).

Video 1. The mLST process of **1b** (or **1a4**) to **1a**.

Video 2. The mLST process of **1b** (or **1a4**) through **1a3**-**1a1** to **1a**.

Video 3. The mLST process of **2a** through **2b** and **2b1**-**2b3** to **2b4**.

Table S1. Reported Crystallographic and DFT (in Italic) Geometric Properties (in Å) of the **Ni-R** Active Site and Biomimetic Complexes

	$r_{Ni–Fe}$	$r_{Ni–H}$	$r_{Fe–H}$	$r_{Ni–S}$	$r_{Ni–S}$	Ref.
[NiFe]hydrogenase Ni–R[20]	2.57	1.58	1.78	2.21	2.54	20
[(dppe)Ni(μ-pdt)(μ-H)Fe(CO)$_3$]BF$_4$[21]	2.613	1.637	1.459	2.209	2.219	21,22
	2.64	*1.87*	*1.56*	*2.23*	*2.23*	
[(dppe)Ni(μ-pdt)(μ-H)Fe(CO)$_2$(PR$_3$)]BF$_4$[9]	2.643	1.90	1.487			9,23
[(dcpe)Ni(μ-pdt)(μ-H)Fe(CO)$_3$]BF$_4$[24]	2.684	1.905	1.535	2.228	2.230	24
[(dppe)Ni(μ-edt)(μ-H)Fe(CO)$_3$]BF$_4$[24]	2.596	1.843	1.58(4)	1.578		24
[(dppv)Ni(pdt)(μ-H)Fe(dppv)(CO)]$^+$	2.646	1.79	1.56			23
[(dppv)Ni(μ-adt)FeH(CO)2(PPh3)]$^+$	2.651	1.86	1.53			25
[{(dppv)(CO)Fe(pdt)}$_2$(μ-H)Ni]BF$_4$	2.565	1.82	1.65	2.217	2.223	26
	2.584	*1.732*	*1.630*	*2.256*	*2.258*	26
[(R,R-DIPAMP)Ni-(μ-pdt)(H)Fe(CO)3]BAr	2.659	1.94	1.58	2.215	2.217	26
[(X')Ni(μ-H)Fe(MeCN){P(OEt)$_3$}$_3$]BPh$_4$[27]	2.793	2.16	1.57			27
[(X')Ni(μ-H)Fe(dppe)(CO)]PF$_6$[28]	2.688	2.04	1.47	2.173	2.198	28
	2.62		*1.56*	*2.23*	*2.24*	28

dppe = 1,2-Bis(diphenylphosphino)ethane
dcpe = Bis(dicyclohexylphosphino)ethane
dppv = *cis*-1,2-C2H2(PPh2)2
DIPAMP = (CH2P(C6H4-2-OMe)2)2
X' = N,N'-diethyl-3,7-diazanonane-1,9-dithiolato

Table S2. The Experimental (**Ni-R**) and the CCSD and DFT Fully Optimized Geometric Parameters (in Å) (**1a**) for the **Ni-R** Active Site Model **1**

	X-ray	CCSD	BP86		TPSS		B3LYP		M06			LRC[a]	
		BS1[b]	BS2[c]	BS3[d]	BS2	BS3	BS2	BS3	BS2	BS3	BS2[e]	BS2	BS3
$r_{Ni–Fe}$	2.57	2.418	2.630	2.569	2.614	2.566	2.711	2.728	2.670	2.614	2.667	2.599	2.595
$r_{Ni–H}$	1.58	1.514	1.574	1.592	1.579	1.590	1.546	1.544	1.559	1.573	1.574	1.533	1.548
$r_{Fe–H}$	1.78	1.667	1.652	1.681	1.646	1.678	1.676	1.688	1.666	1.687	1.662	1.651	1.676
$r_{Fe–CO}$	1.75	1.753	1.730	1.733	1.737	1.739	1.744	1.752	1.749	1.753	1.749	1.775	1.787
$r_{Fe–CN1}$	1.88	1.902	1.890	1.899	1.905	1.911	1.925	1.940	1.924	1.93	1.915	1.931	1.949
$r_{Fe–CN2}$	1.91	1.912	1.895	1.905	1.903	1.916	1.932	1.950	1.930	1.938	1.918	1.937	1.957
$r_{C=O}$	1.15	1.191	1.193	1.176	1.189	1.172	1.174	1.155	1.169	1.151	1.166	1.144	1.128
$r_{C=N1}$	1.17	1.218	1.197	1.182	1.192	1.178	1.181	1.166	1.180	1.165	1.180	1.164	1.153
$r_{C=N2}$	1.15	1.207	1.196	1.181	1.194	1.177	1.182	1.165	1.181	1.164	1.181	1.164	1.152
$r_{Ni–S(Cys84)}$	2.21	2.238	2.187	2.192	2.179	2.187	2.204	2.202	2.190	2.189	2.187	2.196	2.205
$r_{Ni–S(Cys549)}$	*2.54*	*2.923*	*3.166*	*2.688*	*3.658*	*2.714*	*3.770*	*3.380*	*3.713*	*2.915*	*3.705*	*2.953*	*2.851*
$r_{Ni–S(Cys81)}$	2.24	2.272	2.250	2.251	2.245	2.244	2.274	2.268	2.243	2.254	2.221	2.242	2.247
$r_{Ni–S(Cys546)}$	2.18	2.297	2.168	2.170	2.192	2.171	2.243	2.212	2.222	2.199	2.223	2.212	2.211
$r_{Fe–S(Cys84)}$	2.26	2.373	2.267	2.280	2.262	2.273	2.313	2.307	2.294	2.295	2.280	2.321	2.327
$r_{Fe–S(Cys549)}$	2.31	2.547	2.418	2.407	2.393	2.397	2.428	2.444	2.392	2.416	2.383	2.393	2.394

[a] Long range corrected functional: LC-ωPBE. [b] Basis set 1 (BS1): 6-31G** (Fe, Ni, S, H) and STO-3G (C, N, O and group CH_3). [c] BS2: DZVP2. [d] BS3: Def2-TZVP. [e] In the diethylamine solution.

Table S3. Experimental (**Ni-R**) and DFT/DZVP2 Constraint Optimized Bond Length (in Å) and Angle (in degree) for **1a1** with a fixed $r_{Ni–S(4)}$ bond distance of 2.539 Å and the Relative Total electronic Energy ΔE_e (in kcal/mol) between **1a** and **1a1**

	X-ray	CCSD	BP86		TPSS		B3LYP		M06		LRC[b]	
		BS1	BS2	BS3	BS2	BS3	BS2	BS3	BS2	BS3	BS2	BS3
$r_{Ni–Fe}$	2.57	2.365	2.541	2.553	2.525	2.542	2.579	2.590	2.557	2.558	2.532	2.548
$r_{Ni–H}$	1.58	1.515	1.597	1.601	1.595	1.597	1.569	1.572	1.590	1.596	1.555	1.564
$r_{Fe–H}$	1.78	1.690	1.664	1.684	1.659	1.679	1.665	1.688	1.669	1.688	1.653	1.674
$r_{Fe–CO}$	1.75	1.742	1.734	1.735	1.739	1.740	1.747	1.753	1.751	1.754	1.774	1.784
$r_{Fe–CN1}$	1.88	1.908	1.886	1.900	1.898	1.910	1.920	1.938	1.918	1.928	1.931	1.949
$r_{Fe–CN2}$	1.91	1.909	1.891	1.905	1.903	1.916	1.927	1.946	1.925	1.937	1.936	1.955
$r_{C=O}$	1.15	1.192	1.195	1.176	1.190	1.173	1.175	1.156	1.169	1.151	1.144	1.129
$r_{C=N1}$	1.17	1.213	1.197	1.182	1.193	1.178	1.182	1.166	1.181	1.165	1.164	1.153
$r_{C=N2}$	1.15	1.208	1.196	1.181	1.192	1.177	1.181	1.165	1.180	1.164	1.164	1.152
$r_{Ni–S(Cys84)}$	2.21	2.265	2.204	2.196	2.198	2.193	2.235	2.228	2.212	2.199	2.216	2.220
$r_{Ni–S(Cys549)}$[e]	2.54	**2.539**	**2.539**	**2.539**	**2.539**	**2.539**	**2.539**	**2.539**	**2.539**	**2.539**	**2.539**	**2.539**
$r_{Ni–S(Cys81)}$	2.24	2.328	2.252	2.251	2.247	2.247	2.284	2.283	2.262	2.262	2.249	2.255
$r_{Ni–S(Cys546)}$	2.18	2.271	2.189	1.176	2.196	2.183	2.262	2.245	2.234	2.218	2.227	2.225
$r_{Fe–S(Cys84)}$	2.26	2.398	2.296	2.277	2.296	2.279	2.437	2.344	2.332	2.310	2.338	2.338
$r_{Fe–S(Cys549)}$	2.31	2.532	2.419	2.402	2.405	2.390	2.357	2.424	2.413	2.400	2.381	2.380
$\theta_{S(5)-Ni-S(4)}$	107.9	107.2	119.1	118.8	117.9	117.4	114.7	114.2	114.9	114.2	113.7	113.3
ΔE_e		0.710	1.606	0.270	2.375	0.296	5.687	3.324	3.871	1.349	1.490	1.200

[a] Long range corrected functional: LC-ωPBE. [b] Basis set 1 (BS1): 6-31G** (Fe, Ni, S, H) and STO-3G (C, N, O and group CH_3). [c] BS2: DZVP2. [d] BS2: Def2-TZVP. [e] Constraint $r_{Ni–S(Cys549)/S(4)}$ (in Bold) in the geometry optimizations.

Table S4. M06/DZVP2 Geometric Parameters (in ° and Å), QTAIM Topological Properties (Charge Q and Electron Delocalization Index δ in atomic unit), CO vibrational frequency (in cm^{-1}), and Relative Total Electronic Energies ΔE_e (kcal/mol) for the **Ni-R** Active Site Models **1a** and **1b**

	**Ni-R**[a]	**1a**[b]	**1a**[c]	**1b**[c]	**1a1**[d]	**1a2**[d]	**1a3**[d]	**1a4**[d]
$\theta_{S5-Ni-S4}$	107.9	138.0	139.3	166.1	114.9	**140.0**	**160.0**	**180.0**
r_{Ni-Fe}	2.57	2.670	2.667	2.723	2.557	2.604	2.671	2.724
r_{Ni-H}	1.58	1.559	1.574	1.960	1.590	1.671	1.810	1.909
r_{Fe-H}	1.78	1.666	1.662	1.620	1.669	1.647	1.622	1.603
r_{Fe-CO}	1.75	1.749	1.749	1.771	1.751	1.759	1.769	1.774
r_{Fe-CN1}	1.88	1.924	1.915	1.903	1.918	1.915	1.909	1.905
r_{Fe-CN2}	1.91	1.930	1.918	1.900	1.925	1.922	1.916	1.913
$r_{C=O}$	1.15	1.169	1.166	1.168	1.169	1.170	1.171	1.172
$r_{C=N(1)}$	1.17	1.180	1.180	1.182	1.181	1.181	1.182	1.183
$r_{C=N(2))}$	1.15	1.181	1.181	1.181	1.180	1.180	1.180	1.180
$r_{Ni-S((3)}$	2.21	2.190	2.187	2.234	2.212	**2.212**	**2.212**	**2.212**
$r_{Ni-S(4)}$	2.54	3.713	3.705	2.302	**2.539**	**2.539**	**2.539**	**2.539**
$r_{Ni-S(5)}$	2.24	2.243	2.221	2.265	2.262	2.267	2.265	2.265
$r_{Ni-S(6)}$	2.18	2.222	2.223	2.257	2.234	2.229	2.236	2.253
$r_{Fe-S(3)}$	2.26	2.294	2.280	2.374	2.332	2.330	2.355	2.413
$r_{Fe-S(4)}$	2.31	2.392	2.383	2.401	2.413	2.418	2.445	2.431
Q_{Ni}					0.392	0.398	0.399	0.401
Q_{Fe}					0.834	0.826	0.820	0.821
Q_H					-0.215	-0.252	-0.281	-0.298
δ_{Ni-H}					0.544	0.462	0.342	0.261
δ_{Fe-H}					0.408	0.465	0.551	0.609
$\delta_{Ni-S(3)}$					0.772	0.799	0.817	0.821
$\delta_{Ni-S(4)}$					0.370	0.409	0.478	0.533
$\delta_{Ni-S(5)}$					0.774	0.788	0.799	0.789
$\delta_{Ni-S(6)}$					0.636	0.644	0.643	0.636
$\delta_{Fe-S(3)}$					0.608	0.602	0.576	0.533
$\delta_{Fe-S(4)}$					0.599	0.588	0.548	0.533
ν_{CO}		2023			2026			2003
ΔE_e			0.00[e]	8.28[e]	0.00[f]	3.69[f]	8.05[f]	12.97[f]

[a] X-ray structure. [b] Fully Optimized in the gas phase. [c] Optimization in the diethylamine solution. [d] Constrained $\theta_{S(5)-Ni-S(4)}$, $r_{Ni-S(3)}$, and $r_{Ni-S(4)}$ (in Bold) in the geometry optimizations. [e] Relative to **1a** in the solution. [f] Relative to **1b** in the gas phase.

Table S5. M06/DZVP2 Geometric Parameters (in ° and Å), QTAIM Topological Properties (Charge Q and Electron Delocalization Index δ in atomic unit), and Relative Total Electron Energies ΔE_e (kcal/mol) in the Gas Phass, Relative Gibbs Free Energy ΔG (kcal/mol) in the Acetonitrile Solution, Hydride and Hydrogen Bonding Free Energy ΔG_H^- and $\Delta G_H^\bullet$ (kcal/mol) in the Acetonitrile Solution for the Biomimetic Models **2a** and **2b**[a]

M06/DZVP2	**2a***[a]	**2a***[b]	**2a**[c]	**2b**[d,e]	**2b1**[d.e]	**2b2**[d.e]	**2b3**[d,e]	**2b4**[d.e]
$\theta_{P5–Ni–S4}$	175.3	178.8	177.3	175.9	**165.3**	**145.3**	**125.3**	**105.3**
$r_{Ni–Fe}$	2.614	2.649	2.657	2.665	2.636	2.58	2.49	2.421
$r_{Ni–H}$	1.638	1.917	1.922	1.897	1.856	1.784	1.678	1.615
$r_{Fe–H}$	1.460	1.580	1.583	1.588	1.597	1.614	1.648	1.674
$r_{Fe–CO(ax)}$	1.798	1.827	1.831	1.837	1.835	1.831	1.822	1.817
$r_{Fe–CO(eq)}$	1.802	1.801	1.802	1.801	1.802	1.806	1.813	1.819
$r_{Fe–CO(eq)}$	1.786	1.801	1.802	1.801	1.802	1.804	1.806	1.81
$r_{C=O(ax)}$	1.141	1.149	1.147	1.147	1.147	1.147	1.147	1.146
$r_{C=O(eq)}$	1.133	1.148	1.147	1.147	1.147	1.146	1.145	1.144
$r_{C=O(eq)}$	1.142	1.148	1.147	1.147	1.147	1.147	1.146	1.146
$r_{Ni-S(4)}$	2.219	2.249	2.248	2.246	**2.246**	**2.246**	**2.246**	**2.246**
$r_{Ni-S(3)}$	2.210	2.234	2.236	2.233	**2.233**	**2.233**	**2.233**	**2.233**
$r_{Ni-P(6)}$	2.160	2.183	2.175	2.172	2.172	2.172	2.172	2.172
$r_{Ni-P(5)}$	2.173	2.183	2.175	2.172	2.172	2.172	2.172	2.172
$r_{Fe-S(4)}$	2.321	2.360	2.363	2.370	2.381	2.394	2.400	2.406
$r_{Fe-S(3)}$	2.321	2.351	2.352	2.359	2.347	2.334	2.343	2.361
Q_{Ni}				0.190	0.181	0.168	0.159	0.166
Q_{Fe}				0.795	0.797	0.799	0.799	0.799
Q_H				-0.219	-0.218	-0.218	-0.212	-0.210
$\delta_{Ni–H}$				0.251	0.277	0.327	0.413	0.480
$\delta_{Fe–H}$				0.625	0.609	0.578	0.513	0.465
$\delta_{Ni–S(3)}$				0.713	0.714	0.721	0.731	0.725
$\delta_{Ni–S(4)}$				0.697	0.689	0.667	0.622	0.561
$\delta_{Ni–P(5)}$				0.795	0.801	0.810	0.804	0.777
$\delta_{Ni–P(6)}$				0.787	0.787	0.792	0.793	0.791
$\delta_{Fe–S(3)}$				0.623	0.630	0.640	0.646	0.651
$\delta_{Fe–S(4)}$				0.622	0.617	0.619	0.635	0.644
ΔE_e			-3.92	0.00	1.04	6.59	14.35	23.11
ΔG				0.00				21.30
ΔG_H^-	79	72.50	73.03	0.00				0.80
$\Delta G_H^\bullet$	57	48.50	44.02					

[a] Experimental values. [b] R = Ph. [c] R = CH_3. [d] The pdt folding to the Fe site. [e] Constrained $\theta_{P5–Ni–S4}$, Ni–S(4), and Ni–S(3) bonds (in Bold).

Table S6. M06/DZVP2 Geometric Parameters (in ˚ and Å), QTAIM Topological Properties (Charge Q and Electron Delocalization Index δ in atomic unit), and Relative Total Electron Energies ΔE_e (kcal/mol) for the Biomimetic Models **3a** and **6-7**

M06/DZVP2	**3b**	**3c**	**3a**	**3a1**[a]	**3a2**[a]	**3a3**[a]	**6a**	**6b**	**7**
$\theta_{N(5)–Ni–S(4)}$	172.0	177.0	171.4	**160.0**	**140.0**	**120.0**	145.3	138.9	162.8
$r_{Ni–Fe}$	2.746	2.535	2.590	2.517	2.404	2.366	2.504	2.514	2.620
$r_{Ni–H}$	2.264	2.061	2.081	1.972	1.781	1.664	1.685	1.618	1.975
$r_{Fe–H}$	1.586	1.578	1.581	1.593	1.627	1.630	1.610	1.617	1.599
$r_{Fe–Cp}$	1.669	1.662	1.666	1.551	1.633	1.664	1.672	1.677	1.668
$r_{Ni-S(3)}$	2.189	2.180	2.173	2.168	2.204	2.268	2.165	2.160	2.180
$r_{Ni-S(4)}$	2.188	2.185	2.198	2.222	2.297	2.398	2.636	2.804	2.273
$r_{Ni-N(5)}$	1.964	1.931	1.951	1.959	2.009	2.045	2.015	2.003	1.991
$r_{Ni-N(6)}$	1.964	1.933	1.938	1.930	1.963	2.055	1.974	1.989	1.957
$r_{Fe-S(4)}$	2.346	2.451	2.397	2.451	2.521	2.419	2.378	2.375	2.374
$r_{Fe-S(3)}$	2.349	2.426	2.378	2.367	2.400	2.552	2.272	2.268	2.327
Q_{Ni}	0.548	0.540	0.544	0.543	0.524	0.515	0.530		0.548
Q_{Fe}	0.692	0.682	0.680	0.685	0.704	0.733	0.711		0.692
Q_H	-0.349	-0.377	-0.373	-0.363	-0.335	-0.294	-0.261		-0.349
$\delta_{Ni–H}$	0.239	0.189	0.182	0.235	0.372	0.510	0.455		0.239
$\delta_{Fe–H}$	0.678	0.754	0.731	0.702	0.621	0.542	0.561		0.678
$\delta_{Ni–S(3)}$	0.677	0.817	0.796	0.751	0.629	0.440	0.845		0.677
$\delta_{Ni–S(4)}$	0.848	0.824	0.832	0.828	0.796	0.761	0.311		0.848
$\delta_{Ni–N(5)}$	0.518	0.535	0.536	0.542	0.523	0.453	0.498		0.518
$\delta_{Ni–N(6)}$	0.510	0.537	0.528	0.522	0.482	0.451	0.483		0.510
$\delta_{Fe–S(3)}$	0.590	0.538	0.602	0.570	0.543	0.636	0.711		0.590
$\delta_{Fe–S(4)}$	0.631	0.555	0.590	0.599	0.557	0.399	0.627		0.631
ΔE_e	0.08	5.75	0.00	1.31	8.55	20.65	0.00	0.50	

[a] Constrained $\theta_{S5–Ni–S4}$ (in bold).

Table S7. The Calculated and experimental Hydride Bonding Energy ΔE_{H^-} and Free Energy ΔG_{H^-} (kcal/mol) for **2a** and **2b** at the M06/DZVP2 level in the Gas Phase and MeCN Solution.

	**2a**		**2b**	
	Cal.	Exp.	Cal.	Exp.
ΔE_{H^-} (gas)	-84.36		-85.99	
ΔG_{H^-} (gas)	-295.14		-313.90	
ΔG_{H^-} (MeCN)	-72.50	-79	-73.03	

Table S8. M06/DZVP2 Geometric Parameters (in ˚ and Å) and QTAIM Topological Properties (Charge Q and Electron Delocalization Index δ in atomic unit) for the Biomimetic Model **3a-b** and **4a-c**

M06/DZVP2	**4a**	**4b**	**5a**	**5b**	**5c**
$\theta_{P5–Ni–S4}$	103.8	98.6	101.0	113.1	120.7
$r_{Ni–Fe}$	2.506	2.488	2.491	2.508	2.519
$r_{Ni–H}$	1.616	1.628	1.615	1.637	1.659
$r_{Fe–H}$	1.692	1.685	1.689	1.666	1.649
$r_{Fe–CO(ax)}$	1.811	1.808	1.810	1.815	1.814
$r_{Fe–CO(eq)}$	1.828	1.826	1.824	1.823	1.821
$r_{Fe–CO(eq)}$	1.816	1.821	1.822	1.813	1.814
$r_{C=O(ax)}$	1.146	1.146	1.146	1.146	1.146
$r_{C=O(eq)}$	1.144	1.144	1.144	1.144	1.145
$r_{C=O(eq)}$	1.145	1.145	1.145	1.146	1.146
$r_{Ni-S(4)}$	2.614	2.491	2.425	2.487	2.508
$r_{Ni-S(3)}$	2.195	2.210	2.233	2.202	2.196
$r_{Ni-P(6)}$	2.174	2.177	2.182	2.179	2.178
$r_{Ni-P(5)}$	2.116	2.120	2.133	2.124	2.138
$r_{Fe-S(4)}$	2.335	2.317	2.358	2.358	2.361
$r_{Fe-S(3)}$	2.343	2.359	2.305	2.329	2.313
Q_{Ni}	0.189	0.176	0.169	0.170	0.177
Q_{Fe}	0.812	0.805	0.805	0.805	0.799
Q_{H}	-0.246	-0.244	-0.234	-0.231	-0.230
$\delta_{Ni–H}$	0.488	0.472	0.479	0.451	0.428
$\delta_{Fe–H}$	0.456	0.461	0.443	0.476	0.497
