# Supplementary material for: How Geometric Constraints Control the Hydride Position and Activity in [NiFe]-Hydrogenases and Their Biomimetic Complexes
Source: Inorg Chem. 2025 May 9;64(20):10078–86. doi: 10.1021/acs.inorgchem.5c00670 (PMC12117559; doi:10.1021/acs.inorgchem.5c00670)
Supplement: Supplementary file 2 [file ic5c00670_si_002.pdf]

Supporting Information II

How Geometric Constraints Control the Hydride Position and Activity in [NiFe]-Hydrogenases and Their Biomimetic Complexes

Shuqiang Niu* and Michael B. Hall*

Department of Chemistry, Texas A&M University, College Station, TX 77843-3257

*To whom correspondence may be addressed. E-mail: sn72@tamu.edu and MBHall@tamu.edu

Cartesian coordinates of calculated models at the M06/DZVP2 level

1a

Fe(1)	-1.528	0.225	-0.154
Ni(2)	1.046	-0.395	0.191
C(3)	-2.581	-1.374	-0.345
N(4)	-3.142	-2.406	-0.448
C(5)	-1.015	0.095	-2.011
N(6)	-0.648	-0.013	-3.128
C(7)	-2.898	1.262	-0.476
O(8)	-3.819	1.951	-0.685
S(9)	0.213	1.622	0.374
C(10)	0.935	2.433	-1.106
H(11)	2.015	2.541	-0.961
H(12)	0.470	3.418	-1.212
H(13)	0.741	1.836	-2.000
S(14)	-2.009	0.290	2.188
C(15)	-1.614	-1.405	2.742
H(16)	-0.540	-1.612	2.631
H(17)	-2.172	-2.142	2.154
H(18)	-1.884	-1.511	3.800
S(19)	3.242	0.045	0.301
C(20)	3.410	1.308	1.614
H(21)	4.475	1.465	1.821
H(22)	2.911	0.983	2.530
H(23)	2.962	2.259	1.310
S(24)	1.421	-2.551	-0.196
C(25)	0.239	-3.025	-1.504
H(26)	0.517	-3.999	-1.914
H(27)	0.194	-2.244	-2.268
H(28)	-0.746	-3.072	-1.027
H(29)	2.529	-2.462	-0.967
H(30)	-0.413	-0.938	0.268

1a1

Fe(1)	-1.412	0.419	-0.325
Ni(2)	1.035	-0.184	0.108
C(3)	-2.406	-1.219	-0.391
N(4)	-2.944	-2.270	-0.426
C(5)	-1.720	0.704	-2.204
N(6)	-1.873	0.903	-3.357
C(7)	-2.807	1.374	0.132
O(8)	-3.741	2.005	0.442
S(9)	0.384	1.903	-0.228
C(10)	0.990	2.303	-1.901
H(11)	2.084	2.293	-1.866
H(12)	0.633	3.297	-2.188
H(13)	0.615	1.574	-2.622
S(14)	-0.701	0.031	1.948
C(15)	-1.249	-1.639	2.440
H(16)	-0.693	-2.425	1.912
H(17)	-2.309	-1.787	2.220
H(18)	-1.071	-1.754	3.516
S(19)	3.164	0.222	0.758
C(20)	2.916	1.168	2.303
H(21)	3.891	1.406	2.746
H(22)	2.319	0.589	3.013
H(23)	2.377	2.096	2.090
S(24)	1.435	-2.381	0.133
C(25)	0.405	-3.173	-1.150
H(26)	0.662	-4.231	-1.251
H(27)	0.516	-2.638	-2.093
H(28)	-0.635	-3.057	-0.824
H(29)	2.609	-2.404	-0.535
H(30)	-0.163	-0.545	-0.872

1a2

Fe(1)	-1.355	0.437	-0.283
Ni(2)	1.122	-0.229	0.166
C(3)	-2.411	-1.155	-0.411
N(4)	-2.996	-2.180	-0.461
C(5)	-1.655	0.828	-2.140
N(6)	-1.812	1.101	-3.277
C(7)	-2.717	1.424	0.232
O(8)	-3.632	2.068	0.573
S(9)	0.471	1.870	-0.085
C(10)	1.091	2.342	-1.734
H(11)	2.185	2.317	-1.702
H(12)	0.733	3.350	-1.966
H(13)	0.710	1.650	-2.486

S(14)	-0.653	-0.072	1.974
C(15)	-1.229	-1.748	2.409
H(16)	-0.619	-2.537	1.949
H(17)	-2.261	-1.898	2.081
H(18)	-1.161	-1.858	3.497
S(19)	3.352	0.073	-0.110
C(20)	3.808	1.281	1.189
H(21)	4.886	1.478	1.150
H(22)	3.549	0.899	2.182
H(23)	3.265	2.221	1.039
S(24)	1.404	-2.437	0.275
C(25)	0.324	-3.223	-0.968
H(26)	0.529	-4.296	-1.022
H(27)	0.464	-2.733	-1.932
H(28)	-0.709	-3.039	-0.651
H(29)	2.576	-2.559	-0.388
H(30)	-0.162	-0.544	-0.856

1a3

Fe(1)	-1.364	0.498	-0.188
Ni(2)	1.176	-0.242	0.183
C(3)	-2.487	-1.044	-0.266
N(4)	-3.096	-2.057	-0.254
C(5)	-1.767	0.955	-2.005
N(6)	-2.003	1.277	-3.116
C(7)	-2.635	1.561	0.429
O(8)	-3.499	2.247	0.822
S(9)	0.547	1.866	-0.045
C(10)	1.003	2.352	-1.740
H(11)	2.058	2.639	-1.771
H(12)	0.362	3.190	-2.028
H(13)	0.817	1.523	-2.423
S(14)	-0.543	-0.071	2.043
C(15)	-1.072	-1.757	2.513
H(16)	-0.222	-2.450	2.560
H(17)	-1.820	-2.133	1.807
H(18)	-1.522	-1.704	3.510
S(19)	3.161	-0.109	-0.899
C(20)	4.155	1.043	0.120
H(21)	5.133	1.201	-0.350
H(22)	4.308	0.650	1.130
H(23)	3.644	2.008	0.210
S(24)	1.419	-2.448	0.457
C(25)	0.219	-3.234	-0.673
H(26)	0.363	-4.318	-0.680
H(27)	0.332	-2.801	-1.667

H(28)	-0.783	-2.974	-0.316
H(29)	2.526	-2.609	-0.306
H(30)	-0.289	-0.526	-0.842

1a4

Fe(1)	-1.422	0.537	-0.041
Ni(2)	1.182	-0.190	0.294
C(3)	-2.557	-0.993	-0.066
N(4)	-3.161	-2.008	-0.010
C(5)	-1.860	0.989	-1.847
N(6)	-2.118	1.312	-2.953
C(7)	-2.649	1.654	0.588
O(8)	-3.496	2.367	0.974
S(9)	0.553	1.919	0.066
C(10)	0.915	2.441	-1.641
H(11)	1.966	2.732	-1.732
H(12)	0.263	3.290	-1.868
H(13)	0.684	1.633	-2.334
S(14)	-0.537	-0.018	2.154
C(15)	-1.052	-1.695	2.665
H(16)	-0.187	-2.339	2.863
H(17)	-1.704	-2.143	1.908
H(18)	-1.621	-1.594	3.595
S(19)	2.716	-0.343	-1.365
C(20)	4.144	0.583	-0.692
H(21)	4.968	0.569	-1.417
H(22)	4.497	0.150	0.250
H(23)	3.864	1.624	-0.498
S(24)	1.517	-2.359	0.804
C(25)	0.370	-3.169	-0.369
H(26)	0.484	-4.255	-0.318
H(27)	0.579	-2.785	-1.368
H(28)	-0.646	-2.864	-0.100
H(29)	2.624	-2.486	0.026
H(30)	-0.412	-0.515	-0.704

2a

Ni(2)	0.191	-0.266	0.654
S(3)	0.774	-1.259	-1.262
S(4)	-1.973	-0.394	0.057
C(5)	-0.141	-2.845	-1.401
C(6)	-1.172	-3.091	-0.316
C(7)	-2.356	-2.145	-0.344
C(8)	-1.743	1.982	-1.668
O(9)	-2.392	2.924	-1.581
C(10)	-1.637	-0.365	-3.039

O(11)	-2.222	-0.904	-3.866
C(12)	0.552	1.252	-2.773
O(13)	1.391	1.722	-3.399
P(14)	2.264	-0.210	1.306
P(15)	-0.320	0.827	2.464
C(16)	2.338	0.472	3.020
C(17)	1.236	1.514	3.176
C(18)	3.292	0.903	0.292
C(19)	3.197	-1.771	1.373
C(20)	-1.053	-0.193	3.783
C(21)	-1.440	2.249	2.290
H(22)	0.079	1.226	-0.553
H(23)	-0.594	-2.882	-2.397
H(24)	0.634	-3.613	-1.365
H(25)	-1.552	-4.112	-0.437
H(26)	-0.687	-3.044	0.668
H(27)	-3.091	-2.440	0.408
H(28)	-2.858	-2.164	-1.317
H(29)	2.181	-0.368	3.707
H(30)	3.330	0.882	3.230
H(31)	1.472	2.420	2.606
H(32)	1.090	1.812	4.219
H(33)	4.321	0.923	0.661
H(34)	3.288	0.546	-0.741
H(35)	2.875	1.912	0.310
H(36)	4.182	-1.608	1.819
H(37)	2.646	-2.504	1.966
H(38)	3.318	-2.157	0.360
H(39)	-0.399	-1.037	4.012
H(40)	-1.217	0.400	4.687
H(41)	-2.009	-0.587	3.429
H(42)	-1.018	2.960	1.576
H(43)	-2.409	1.908	1.917
H(44)	-1.580	2.743	3.255

2b

Fe(1)	-0.691	0.542	-1.753
Ni(2)	0.197	-0.235	0.638
S(3)	0.779	-1.240	-1.270
S(4)	-1.960	-0.372	0.027
C(5)	-0.084	-2.865	-1.308
C(6)	-1.597	-2.849	-1.386
C(7)	-2.305	-2.162	-0.236
C(8)	-1.704	2.028	-1.656
O(9)	-2.339	2.977	-1.542
C(10)	-1.591	-0.188	-3.178

O(11)	-2.141	-0.548	-4.117
C(12)	0.610	1.305	-2.737
O(13)	1.474	1.786	-3.318
P(14)	2.259	-0.227	1.320
P(15)	-0.330	0.824	2.460
C(16)	2.315	0.418	3.050
C(17)	1.223	1.470	3.216
C(18)	3.328	0.884	0.347
C(19)	3.160	-1.807	1.372
C(20)	-1.102	-0.217	3.741
C(21)	-1.428	2.265	2.300
H(22)	0.101	1.231	-0.562
H(23)	0.329	-3.390	-2.172
H(24)	0.255	-3.389	-0.407
H(25)	-1.923	-2.425	-2.338
H(26)	-1.934	-3.892	-1.401
H(27)	-2.059	-2.646	0.716
H(28)	-3.386	-2.228	-0.373
H(29)	2.137	-0.436	3.715
H(30)	3.309	0.810	3.284
H(31)	1.481	2.387	2.674
H(32)	1.065	1.742	4.264
H(33)	4.351	0.875	0.733
H(34)	3.334	0.548	-0.693
H(35)	2.933	1.902	0.379
H(36)	4.141	-1.673	1.835
H(37)	2.586	-2.540	1.943
H(38)	3.288	-2.178	0.353
H(39)	-0.457	-1.068	3.971
H(40)	-1.286	0.360	4.652
H(41)	-2.050	-0.599	3.356
H(42)	-0.985	2.987	1.611
H(43)	-2.394	1.944	1.902
H(44)	-1.581	2.739	3.274

2b1

Fe(1)	-0.586	0.619	-1.718
Ni(2)	0.261	-0.182	0.646
S(3)	0.843	-1.186	-1.262
S(4)	-1.896	-0.319	0.035
C(5)	-0.039	-2.797	-1.360
C(6)	-1.552	-2.766	-1.423
C(7)	-2.235	-2.107	-0.243
C(8)	-1.582	2.116	-1.595
O(9)	-2.207	3.069	-1.467
C(10)	-1.493	-0.060	-3.161

O(11)	-2.046	-0.394	-4.109
C(12)	0.735	1.387	-2.673
O(13)	1.611	1.868	-3.235
P(14)	2.227	-0.523	1.504
P(15)	-0.267	0.877	2.468
C(16)	2.218	0.038	3.263
C(17)	1.290	1.242	3.388
C(18)	3.557	0.429	0.697
C(19)	2.846	-2.234	1.551
C(20)	-1.282	-0.085	3.637
C(21)	-1.143	2.459	2.285
H(22)	0.223	1.273	-0.506
H(23)	0.364	-3.290	-2.247
H(24)	0.299	-3.361	-0.484
H(25)	-1.887	-2.309	-2.357
H(26)	-1.898	-3.805	-1.467
H(27)	-1.963	-2.607	0.692
H(28)	-3.319	-2.177	-0.354
H(29)	1.852	-0.800	3.867
H(30)	3.234	0.263	3.602
H(31)	1.737	2.128	2.922
H(32)	1.077	1.495	4.432
H(33)	4.526	0.221	1.159
H(34)	3.595	0.157	-0.361
H(35)	3.338	1.497	0.769
H(36)	3.787	-2.284	2.106
H(37)	2.108	-2.882	2.027
H(38)	3.015	-2.580	0.528
H(39)	-0.793	-1.036	3.861
H(40)	-1.437	0.474	4.564
H(41)	-2.248	-0.295	3.173
H(42)	-0.557	3.136	1.660
H(43)	-2.107	2.277	1.804
H(44)	-1.314	2.919	3.262

2b2

Fe(1)	-0.447	0.795	-1.717
Ni(2)	0.337	-0.050	0.592
S(3)	0.919	-1.055	-1.316
S(4)	-1.819	-0.188	-0.019
C(5)	-0.006	-2.626	-1.539
C(6)	-1.518	-2.565	-1.582
C(7)	-2.165	-1.964	-0.354
C(8)	-1.413	2.306	-1.525
O(9)	-2.020	3.264	-1.351
C(10)	-1.374	0.215	-3.185

O(11)	-1.939	-0.062	-4.143
C(12)	0.888	1.576	-2.649
O(13)	1.757	2.067	-3.211
P(14)	1.943	-1.004	1.701
P(15)	-0.190	1.009	2.414
C(16)	1.777	-0.568	3.488
C(17)	1.216	0.846	3.597
C(18)	3.647	-0.529	1.254
C(19)	1.977	-2.825	1.675
C(20)	-1.601	0.328	3.347
C(21)	-0.562	2.782	2.266
H(22)	0.393	1.383	-0.470
H(23)	0.384	-3.049	-2.468
H(24)	0.325	-3.275	-0.721
H(25)	-1.859	-2.046	-2.481
H(26)	-1.882	-3.595	-1.684
H(27)	-1.860	-2.508	0.548
H(28)	-3.251	-2.031	-0.433
H(29)	1.090	-1.295	3.937
H(30)	2.739	-0.672	3.999
H(31)	1.973	1.584	3.306
H(32)	0.896	1.089	4.615
H(33)	4.377	-1.087	1.847
H(34)	3.807	-0.734	0.193
H(35)	3.783	0.542	1.421
H(36)	2.700	-3.199	2.406
H(37)	0.987	-3.224	1.910
H(38)	2.275	-3.167	0.680
H(39)	-1.455	-0.742	3.513
H(40)	-1.696	0.838	4.310
H(41)	-2.517	0.462	2.769
H(42)	0.261	3.296	1.766
H(43)	-1.468	2.899	1.664
H(44)	-0.733	3.226	3.250

2b3

Fe(1)	-0.251	0.986	-1.634
Ni(2)	0.456	0.056	0.564
S(3)	1.038	-0.948	-1.344
S(4)	-1.700	-0.081	-0.046
C(5)	0.036	-2.448	-1.692
C(6)	-1.459	-2.294	-1.858
C(7)	-2.182	-1.753	-0.643
C(8)	-1.175	2.502	-1.299
O(9)	-1.757	3.449	-1.019
C(10)	-1.200	0.606	-3.143

O(11)	-1.785	0.462	-4.119
C(12)	1.123	1.783	-2.508
O(13)	2.008	2.279	-3.039
P(14)	1.506	-1.391	1.799
P(15)	-0.071	1.115	2.386
C(16)	1.151	-1.030	3.576
C(17)	0.997	0.478	3.748
C(18)	3.328	-1.373	1.683
C(19)	1.107	-3.164	1.642
C(20)	-1.758	0.912	3.044
C(21)	0.179	2.920	2.393
H(22)	0.641	1.463	-0.333
H(23)	0.479	-2.849	-2.607
H(24)	0.258	-3.157	-0.889
H(25)	-1.686	-1.688	-2.738
H(26)	-1.867	-3.288	-2.076
H(27)	-2.041	-2.425	0.211
H(28)	-3.254	-1.697	-0.844
H(29)	0.218	-1.547	3.831
H(30)	1.939	-1.446	4.211
H(31)	1.968	0.978	3.654
H(32)	0.581	0.746	4.724
H(33)	3.770	-2.137	2.330
H(34)	3.622	-1.559	0.647
H(35)	3.703	-0.388	1.971
H(36)	1.527	-3.701	2.497
H(37)	0.027	-3.320	1.616
H(38)	1.556	-3.566	0.731
H(39)	-1.987	-0.148	3.177
H(40)	-1.830	1.429	4.006
H(41)	-2.487	1.330	2.348
H(42)	1.195	3.151	2.068
H(43)	-0.523	3.385	1.697
H(44)	0.010	3.330	3.392

2b4

Fe(1)	-0.050	1.122	-1.539
Ni(2)	0.603	0.124	0.567
S(3)	1.185	-0.880	-1.341
S(4)	-1.553	-0.013	-0.043
C(5)	0.120	-2.304	-1.804
C(6)	-1.345	-2.046	-2.082
C(7)	-2.169	-1.505	-0.928
C(8)	-0.910	2.651	-1.091
O(9)	-1.447	3.599	-0.735
C(10)	-1.035	0.914	-3.052

O(11)	-1.654	0.887	-4.016
C(12)	1.356	1.904	-2.390
O(13)	2.252	2.389	-2.910
P(14)	0.967	-1.637	1.787
P(15)	0.076	1.183	2.389
C(16)	0.416	-1.292	3.515
C(17)	0.662	0.182	3.823
C(18)	2.728	-2.094	1.968
C(19)	0.176	-3.253	1.478
C(20)	-1.684	1.485	2.757
C(21)	0.838	2.822	2.634
H(22)	0.891	1.510	-0.210
H(23)	0.602	-2.689	-2.706
H(24)	0.244	-3.065	-1.032
H(25)	-1.440	-1.395	-2.954
H(26)	-1.792	-2.999	-2.390
H(27)	-2.283	-2.271	-0.153
H(28)	-3.173	-1.263	-1.283
H(29)	-0.655	-1.526	3.566
H(30)	0.932	-1.960	4.211
H(31)	1.734	0.382	3.932
H(32)	0.169	0.504	4.746
H(33)	2.844	-2.969	2.614
H(34)	3.145	-2.310	0.981
H(35)	3.281	-1.251	2.389
H(36)	0.229	-3.828	2.407
H(37)	-0.871	-3.139	1.193
H(38)	0.711	-3.811	0.708
H(39)	-2.232	0.544	2.830
H(40)	-1.747	2.025	3.706
H(41)	-2.147	2.083	1.969
H(42)	1.917	2.746	2.491
H(43)	0.440	3.520	1.892
H(44)	0.625	3.209	3.634

3a

Fe(1)	-0.003	0.095	-1.814
Ni(2)	-0.370	-0.043	0.746
S(3)	-1.168	-1.601	-0.583
S(4)	-1.367	1.478	-0.442
N(5)	0.855	-1.239	1.655
N(6)	0.187	1.205	2.138
H(7)	1.103	0.247	-0.695
C(8)	0.225	1.375	-3.404
C(9)	-0.822	0.435	-3.701
C(10)	-0.254	-0.860	-3.631

C(11)	1.134	-0.748	-3.299
C(12)	1.422	0.651	-3.174
H(13)	0.114	2.450	-3.342
H(14)	-1.857	0.671	-3.906
H(15)	-0.800	-1.788	-3.732
H(16)	1.837	-1.564	-3.196
H(17)	2.379	1.081	-2.911
C(18)	-0.277	2.783	0.290
C(19)	-0.239	2.589	1.793
C(20)	1.169	-2.360	0.733
C(21)	-0.081	-2.932	0.106
C(22)	1.658	1.059	2.174
C(23)	2.057	-0.410	1.919
C(24)	-0.431	0.770	3.413
C(25)	0.125	-0.552	3.936
C(26)	0.225	-1.685	2.917
H(27)	0.713	2.691	-0.167
H(28)	-0.681	3.770	0.052
H(29)	0.417	3.313	2.303
H(30)	-1.251	2.719	2.190
H(31)	2.058	1.675	1.366
H(32)	2.056	1.443	3.125
H(33)	2.672	-0.451	1.021
H(34)	2.639	-0.834	2.750
H(35)	-1.507	0.697	3.230
H(36)	-0.265	1.545	4.179
H(37)	-0.514	-0.884	4.758
H(38)	1.110	-0.379	4.384
H(39)	-0.767	-2.075	2.670
H(40)	0.819	-2.503	3.355
H(41)	1.774	-1.926	-0.070
H(42)	1.760	-3.125	1.263
H(43)	0.206	-3.614	-0.699
H(44)	-0.677	-3.506	0.824

3a1

Fe(1)	-0.217	0.098	-1.794
Ni(2)	-0.102	0.106	0.720
S(3)	-1.490	-1.327	-0.259
S(4)	-1.000	1.781	-0.324
N(5)	0.899	-1.299	1.584
N(6)	0.716	1.218	2.110
H(7)	1.074	0.058	-0.862
C(8)	0.321	1.129	-3.480
C(9)	-1.072	0.776	-3.557
C(10)	-1.174	-0.630	-3.486

C(11)	0.152	-1.170	-3.364
C(12)	1.068	-0.078	-3.381
H(13)	0.727	2.131	-3.501
H(14)	-1.900	1.471	-3.598
H(15)	-2.093	-1.198	-3.447
H(16)	0.409	-2.219	-3.291
H(17)	2.142	-0.151	-3.281
C(18)	0.399	2.843	0.263
C(19)	0.532	2.656	1.764
C(20)	0.904	-2.462	0.663
C(21)	-0.499	-2.818	0.215
C(22)	2.137	0.803	2.051
C(23)	2.248	-0.714	1.759
C(24)	0.108	0.900	3.419
C(25)	0.455	-0.500	3.910
C(26)	0.274	-1.618	2.888
H(27)	1.304	2.547	-0.276
H(28)	0.185	3.891	0.036
H(29)	1.352	3.248	2.199
H(30)	-0.399	2.979	2.240
H(31)	2.594	1.352	1.226
H(32)	2.660	1.089	2.976
H(33)	2.778	-0.853	0.817
H(34)	2.803	-1.247	2.543
H(35)	-0.974	1.017	3.298
H(36)	0.451	1.633	4.167
H(37)	-0.175	-0.722	4.775
H(38)	1.483	-0.511	4.289
H(39)	-0.788	-1.801	2.704
H(40)	0.722	-2.542	3.288
H(41)	1.474	-2.145	-0.217
H(42)	1.412	-3.315	1.141
H(43)	-0.431	-3.494	-0.642
H(44)	-1.054	-3.345	1.000

3a2

Fe(1)	-0.293	0.111	-1.843
Ni(2)	0.032	0.129	0.538
S(3)	-1.442	-1.455	-0.236
S(4)	-1.107	1.797	-0.342
N(5)	0.983	-1.283	1.516
N(6)	0.450	1.152	2.216
H(7)	1.048	0.162	-0.923
C(8)	-0.076	1.205	-3.566
C(9)	-1.345	0.530	-3.571
C(10)	-1.102	-0.861	-3.472

C(11)	0.313	-1.064	-3.405
C(12)	0.942	0.221	-3.476
H(13)	0.074	2.276	-3.584
H(14)	-2.316	1.006	-3.598
H(15)	-1.856	-1.629	-3.364
H(16)	0.818	-2.019	-3.334
H(17)	2.004	0.412	-3.418
C(18)	0.160	2.889	0.446
C(19)	0.168	2.589	1.942
C(20)	1.139	-2.379	0.538
C(21)	-0.221	-2.828	-0.003
C(22)	1.902	0.870	2.326
C(23)	2.226	-0.593	1.903
C(24)	-0.307	0.660	3.380
C(25)	0.152	-0.721	3.821
C(26)	0.225	-1.748	2.701
H(27)	1.135	2.704	-0.017
H(28)	-0.102	3.940	0.286
H(29)	0.879	3.221	2.495
H(30)	-0.832	2.797	2.336
H(31)	2.408	1.551	1.639
H(32)	2.268	1.094	3.340
H(33)	2.872	-0.578	1.022
H(34)	2.753	-1.139	2.699
H(35)	-1.363	0.647	3.089
H(36)	-0.190	1.363	4.221
H(37)	-0.548	-1.092	4.574
H(38)	1.119	-0.646	4.332
H(39)	-0.783	-1.995	2.358
H(40)	0.701	-2.665	3.083
H(41)	1.739	-1.980	-0.285
H(42)	1.675	-3.227	0.997
H(43)	-0.057	-3.334	-0.958
H(44)	-0.686	-3.558	0.668

3a3

Fe(1)	-0.203	0.179	-1.875
Ni(2)	0.214	0.242	0.453
S(3)	-1.297	-1.407	-0.412
S(4)	-1.012	2.001	-0.284
N(5)	0.991	-1.358	1.482
N(6)	0.047	0.864	2.394
H(7)	1.129	0.217	-0.937
C(8)	-0.286	1.394	-3.556
C(9)	-1.315	0.406	-3.599
C(10)	-0.692	-0.879	-3.561

C(11)	0.715	-0.685	-3.490
C(12)	0.964	0.730	-3.486
H(13)	-0.440	2.462	-3.482
H(14)	-2.378	0.598	-3.626
H(15)	-1.208	-1.828	-3.508
H(16)	1.463	-1.464	-3.435
H(17)	1.931	1.206	-3.406
C(18)	-0.038	2.922	0.981
C(19)	-0.325	2.303	2.345
C(20)	1.322	-2.292	0.395
C(21)	0.037	-2.697	-0.364
C(22)	1.484	0.692	2.746
C(23)	2.067	-0.639	2.170
C(24)	-0.846	0.125	3.300
C(25)	-0.284	-1.236	3.659
C(26)	0.110	-2.057	2.446
H(27)	1.032	2.908	0.739
H(28)	-0.360	3.969	0.985
H(29)	0.172	2.845	3.166
H(30)	-1.404	2.360	2.516
H(31)	2.021	1.531	2.297
H(32)	1.630	0.765	3.834
H(33)	2.839	-0.412	1.430
H(34)	2.530	-1.247	2.963
H(35)	-1.811	0.031	2.789
H(36)	-0.999	0.710	4.222
H(37)	-1.046	-1.795	4.210
H(38)	0.559	-1.128	4.351
H(39)	-0.794	-2.344	1.907
H(40)	0.627	-2.972	2.777
H(41)	2.013	-1.782	-0.282
H(42)	1.818	-3.194	0.793
H(43)	0.315	-2.960	-1.389
H(44)	-0.400	-3.594	0.086

4

Fe(1)	-0.286	0.932	-1.643
Ni(2)	0.579	0.140	0.572
S(3)	1.162	-0.865	-1.290
S(4)	-1.872	-0.142	-0.294
C(5)	0.286	-2.510	-1.058
C(6)	-1.083	-2.538	-1.726
C(7)	-2.218	-1.784	-1.039
C(8)	-1.332	2.405	-1.457
O(9)	-1.993	3.323	-1.282
C(10)	-1.114	0.388	-3.159

O(11)	-1.664	0.058	-4.108
C(12)	1.089	1.805	-2.476
O(13)	1.952	2.358	-2.983
P(14)	0.989	-1.701	1.531
P(15)	0.242	1.050	2.517
C(16)	0.125	-1.655	3.155
C(17)	0.448	-0.295	3.783
C(18)	2.709	-2.191	1.862
C(19)	0.271	-2.977	0.415
C(20)	-1.438	1.700	2.792
C(21)	1.347	2.386	3.073
H(22)	0.397	1.538	-0.218
H(23)	0.936	-3.179	-1.628
H(24)	-0.962	-2.191	-2.756
H(25)	-1.394	-3.587	-1.810
H(26)	-2.619	-2.369	-0.205
H(27)	-3.041	-1.665	-1.749
H(28)	-0.948	-1.740	2.944
H(29)	0.412	-2.487	3.803
H(30)	1.492	-0.265	4.116
H(31)	-0.174	-0.098	4.661
H(32)	2.747	-3.173	2.340
H(33)	3.255	-2.221	0.916
H(34)	3.189	-1.453	2.509
H(35)	0.811	-3.921	0.535
H(36)	-0.756	-3.151	0.750
H(37)	-2.171	0.906	2.633
H(38)	-1.535	2.098	3.806
H(39)	-1.641	2.496	2.071
H(40)	2.387	2.067	2.978
H(41)	1.197	3.263	2.440
H(42)	1.139	2.655	4.113

5

Fe(1)	-0.337	0.971	-1.643
Ni(2)	0.518	0.102	0.526
S(3)	1.278	-0.673	-1.399
S(4)	-1.807	-0.277	-0.285
C(5)	0.576	-2.351	-1.667
C(6)	-0.547	-2.828	-0.752
C(7)	-1.826	-2.004	-0.884
C(8)	-1.523	2.334	-1.416
O(9)	-2.267	3.183	-1.228
C(10)	-1.094	0.306	-3.145
O(11)	-1.573	-0.167	-4.073
C(12)	0.920	2.008	-2.468

O(13)	1.714	2.662	-2.970
P(14)	0.798	-1.733	1.552
P(15)	0.308	1.111	2.443
C(16)	0.078	-1.520	3.234
C(17)	0.540	-0.164	3.767
C(18)	2.496	-2.339	1.809
C(19)	-0.104	-3.093	0.698
C(20)	-1.349	1.807	2.750
C(21)	1.451	2.462	2.875
H(22)	0.356	1.541	-0.217
H(23)	0.247	-2.390	-2.710
H(24)	1.437	-3.022	-1.584
H(25)	-0.817	-3.818	-1.146
H(26)	-2.621	-2.479	-0.301
H(27)	-2.158	-2.017	-1.927
H(28)	-1.012	-1.539	3.115
H(29)	0.359	-2.344	3.896
H(30)	1.608	-0.187	4.010
H(31)	0.006	0.119	4.678
H(32)	2.492	-3.280	2.366
H(33)	2.973	-2.490	0.838
H(34)	3.076	-1.593	2.356
H(35)	-0.985	-3.328	1.305
H(36)	0.546	-3.975	0.720
H(37)	-2.101	1.020	2.665
H(38)	-1.400	2.263	3.743
H(39)	-1.567	2.566	1.994
H(40)	2.483	2.122	2.761
H(41)	1.288	3.300	2.194
H(42)	1.287	2.797	3.903

6

Fe(1)	-0.337	0.971	-1.643
Ni(2)	0.518	0.102	0.526
S(3)	1.278	-0.673	-1.399
S(4)	-1.807	-0.277	-0.285
C(5)	0.576	-2.351	-1.667
C(6)	-0.547	-2.828	-0.752
C(7)	-1.826	-2.004	-0.884
C(8)	-1.523	2.334	-1.416
O(9)	-2.267	3.183	-1.228
C(10)	-1.094	0.306	-3.145
O(11)	-1.573	-0.167	-4.073
C(12)	0.920	2.008	-2.468
O(13)	1.714	2.662	-2.970
P(14)	0.798	-1.733	1.552

P(15)	0.308	1.111	2.443
C(16)	0.078	-1.520	3.234
C(17)	0.540	-0.164	3.767
C(18)	2.496	-2.339	1.809
C(19)	-0.104	-3.093	0.698
C(20)	-1.349	1.807	2.750
C(21)	1.451	2.462	2.875
H(22)	0.356	1.541	-0.217
H(23)	0.247	-2.390	-2.710
H(24)	1.437	-3.022	-1.584
H(25)	-0.817	-3.818	-1.146
H(26)	-2.621	-2.479	-0.301
H(27)	-2.158	-2.017	-1.927
H(28)	-1.012	-1.539	3.115
H(29)	0.359	-2.344	3.896
H(30)	1.608	-0.187	4.010
H(31)	0.006	0.119	4.678
H(32)	2.492	-3.280	2.366
H(33)	2.973	-2.490	0.838
H(34)	3.076	-1.593	2.356
H(35)	-0.985	-3.328	1.305
H(36)	0.546	-3.975	0.720
H(37)	-2.101	1.020	2.665
H(38)	-1.400	2.263	3.743
H(39)	-1.567	2.566	1.994
H(40)	2.483	2.122	2.761
H(41)	1.288	3.300	2.194
H(42)	1.287	2.797	3.903

7

Fe(1)	-0.237	1.044	-1.640
Ni(2)	0.414	0.092	0.587
S(3)	1.128	-0.810	-1.290
S(4)	-1.894	-0.011	-0.335
C(5)	0.252	-2.428	-1.516
C(6)	-1.214	-2.336	-1.921
C(7)	-2.237	-1.687	-1.000
C(8)	-1.217	2.551	-1.404
O(9)	-1.832	3.495	-1.195
C(10)	-1.035	0.623	-3.215
O(11)	-1.557	0.402	-4.211
C(12)	1.196	1.896	-2.379
O(13)	2.103	2.431	-2.827
P(14)	0.927	-1.617	1.739
P(15)	0.131	1.200	2.442
C(16)	0.242	-1.372	3.430

C(17)	0.550	0.067	3.845
C(18)	2.691	-2.009	1.972
C(19)	0.162	-3.122	1.041
C(20)	-1.580	1.734	2.772
C(21)	1.130	2.697	2.719
H(22)	0.449	1.527	-0.200
H(23)	0.768	-2.822	-2.399
H(24)	-1.255	-1.844	-2.897
H(25)	-1.554	-3.363	-2.109
H(26)	-2.426	-2.308	-0.120
H(27)	-3.184	-1.624	-1.541
H(28)	-0.840	-1.534	3.361
H(29)	0.646	-2.103	4.137
H(30)	1.620	0.189	4.051
H(31)	0.010	0.356	4.751
H(32)	2.810	-2.957	2.505
H(33)	3.188	-2.066	1.002
H(34)	3.168	-1.210	2.545
H(35)	0.431	-3.972	1.679
H(36)	-0.922	-2.991	1.118
H(37)	-2.242	0.865	2.788
H(38)	-1.637	2.263	3.728
H(39)	-1.918	2.396	1.971
H(40)	2.186	2.469	2.562
H(41)	0.832	3.462	1.998
H(42)	0.983	3.083	3.731
C(43)	0.602	-3.418	-0.395
H(44)	0.165	-4.381	-0.687
H(45)	1.687	-3.577	-0.407

8

Fe(1)	-0.129	1.073	-1.694
Ni(2)	0.407	0.033	0.537
S(3)	1.105	-0.853	-1.347
S(4)	-1.891	0.095	-0.463
C(5)	0.100	-2.350	-1.720
C(6)	-1.189	-2.630	-0.954
C(7)	-2.242	-1.548	-1.188
C(8)	-1.058	2.618	-1.491
O(9)	-1.644	3.582	-1.292
C(10)	-0.886	0.586	-3.269
O(11)	-1.372	0.244	-4.249
C(12)	1.352	1.867	-2.396
O(13)	2.289	2.368	-2.821
P(14)	1.043	-1.636	1.713
P(15)	0.088	1.154	2.377

C(16)	0.461	-1.352	3.440
C(17)	0.694	0.118	3.782
C(18)	2.831	-1.952	1.846
C(19)	0.297	-3.218	1.161
C(20)	-1.653	1.517	2.773
C(21)	0.932	2.758	2.552
H(22)	0.526	1.495	-0.239
H(23)	-0.132	-2.286	-2.788
H(24)	0.805	-3.179	-1.615
H(25)	-1.584	-3.518	-1.468
H(26)	-3.182	-1.851	-0.715
H(27)	-2.450	-1.441	-2.257
H(28)	-0.608	-1.594	3.464
H(29)	0.969	-2.027	4.136
H(30)	1.764	0.324	3.897
H(31)	0.203	0.407	4.716
H(32)	3.018	-2.849	2.442
H(33)	3.245	-2.086	0.844
H(34)	3.329	-1.098	2.309
H(35)	0.235	-3.876	2.035
H(36)	0.998	-3.693	0.467
H(37)	-2.222	0.586	2.833
H(38)	-1.723	2.059	3.720
H(39)	-2.087	2.122	1.973
H(40)	1.997	2.641	2.344
H(41)	0.513	3.460	1.826
H(42)	0.796	3.161	3.559
C(43)	-1.092	-3.013	0.547
H(44)	-1.621	-2.249	1.135
H(45)	-1.663	-3.934	0.690

9a

Fe(2)	0.429	-0.909	-1.593
S(3)	1.281	1.096	-0.946
S(4)	-1.747	0.044	-1.691
N(5)	-1.885	-0.129	1.380
N(6)	0.035	1.472	2.121
C(7)	2.923	0.769	-0.171
C(8)	2.841	0.134	1.211
C(9)	1.875	-2.364	-1.710
C(10)	1.949	-1.500	-2.841
C(11)	0.714	-1.549	-3.546
C(12)	-0.132	-2.468	-2.845
C(13)	0.570	-2.969	-1.725
C(14)	2.582	1.108	2.359
H(15)	3.470	0.114	-0.854

H(16)	3.462	1.724	-0.128
H(17)	2.081	-0.657	1.182
H(18)	3.796	-0.362	1.426
H(19)	2.656	-2.539	-0.983
H(20)	2.783	-0.855	-3.088
H(21)	0.450	-0.980	-4.425
H(22)	-1.164	-2.678	-3.090
H(23)	0.175	-3.653	-0.986
H(24)	2.556	0.546	3.301
H(25)	3.452	1.771	2.454
C(26)	-0.282	0.473	3.170
C(27)	-1.403	-0.501	2.719
C(28)	-0.928	2.602	2.136
C(29)	-2.361	2.154	2.351
C(30)	-2.820	1.036	1.435
C(31)	-2.559	-1.186	0.650
C(32)	-2.581	-1.158	-0.691
H(33)	0.623	-0.108	3.348
H(34)	-0.532	0.980	4.115
H(35)	-2.230	-0.527	3.445
H(36)	-0.997	-1.511	2.636
H(37)	-0.815	3.129	1.182
H(38)	-0.653	3.301	2.943
H(39)	-2.507	1.870	3.400
H(40)	-3.017	3.014	2.188
H(41)	-2.925	1.385	0.404
H(42)	-3.798	0.668	1.775
H(43)	-3.104	-1.915	1.248
H(44)	-3.164	-1.924	-1.202
C(45)	1.389	2.054	2.285
H(46)	1.516	2.748	1.446
H(47)	1.390	2.661	3.207
H(48)	0.188	-1.167	-0.023
